# Propagation of oestrogen receptor-positive and oestrogen-responsive normal human breast cells in culture

**DOI:** 10.1038/ncomms9786

**Published:** 2015-11-13

**Authors:** Agla J. Fridriksdottir, Jiyoung Kim, René Villadsen, Marie Christine Klitgaard, Branden M. Hopkinson, Ole William Petersen, Lone Rønnov-Jessen

**Affiliations:** 1 Department of Cellular and Molecular Medicine, Faculty of Health Sciences, University of Copenhagen, DK-2200 Copenhagen N, Denmark; 2 Danish Stem Cell Centre, Faculty of Health Sciences, University of Copenhagen, DK-2200 Copenhagen N, Denmark; 3 Department of Biology, University of Copenhagen, DK-2100 Copenhagen Ø, Denmark

## Abstract

Investigating the susceptibility of oestrogen receptor-positive (ER^pos^) normal human breast epithelial cells (HBECs) for clinical purposes or basic research awaits a proficient cell-based assay. Here we set out to identify markers for isolating ER^pos^ cells and to expand what appear to be post-mitotic primary cells into exponentially growing cultures. We report a robust technique for isolating ER^pos^ HBECs from reduction mammoplasties by FACS using two cell surface markers, CD166 and CD117, and an intracellular cytokeratin marker, Ks20.8, for further tracking single cells in culture. We show that ER^pos^ HBECs are released from growth restraint by small molecule inhibitors of TGFβ signalling, and that growth is augmented further in response to oestrogen. Importantly, ER signalling is functionally active in ER^pos^ cells in extended culture. These findings open a new avenue of experimentation with normal ER^pos^ HBECs and provide a basis for understanding the evolution of human breast cancer.

## Abstract (web summary)

Culturing normal primary breast cells that express the oestrogen receptor is difficult. Here, the authors isolate oestrogen receptor positive normal breast cells using the cell surface markers CD166 and CD117, and show that the cultures can be repeatedly passaged and retain oestrogen receptor protein expression.

## Graphical abstract

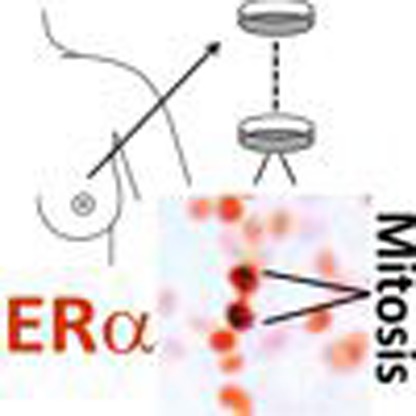

Understanding the taxonomy and evolution of breast cancer has always relied heavily on the use of normal cell types as reference (for review see ref. 1). Nevertheless, ever since the first protocol for cultivation of normal human breast epithelial cells appeared three decades ago^2^, it has become increasingly clear that there are no protocols that support propagation of oestrogen receptor-positive (ER^pos^) cells. Thus, along with the appreciation of epithelial cell lineages in the human breast, primarily the luminal lineage and the basal/myoepithelial lineage, it became evident that the fastest growing cells in culture are of basal origin^3^,^4^. Moreover, when it was revealed that ER^pos^ cells *in situ* accounted for an average of about 7% (mean 6.6%, ranging from 1.2 to 19.1% in a series of 15 normal breast samples) of the cells within the luminal epithelial lineage^5^, the chances of recovering these cells in culture without prospective isolation would in many cases be elusive. Thus, in culture medium that allowed luminal cells to be maintained after passaging, endogenous ER expression disappeared^6^,^7^. Likewise, even when employing freshly isolated small pieces of breast tissue, including the surrounding stroma thus preserving tissue architecture, steroid receptor expression is eventually lost^8^,^9^. As a consequence of this, the comparison of cancer with ‘normal', for example, the HMT-3522, MCF10A and 184B5 cell lines^1^,^6^,^10^, in cell-based assays has relied on normal cells lacking ER expression.

In an attempt to overcome the loss of receptor expression, ER has been ectopically introduced into such cell lines. This approach, however, has had a number of shortcomings, for example, instead of responding to oestrogen by increased proliferation as expected, the ER-transfected cells under standard culture conditions show growth inhibition^11^,^12^. Accordingly, most of our current knowledge of ER expression, regulation and action comes from breast carcinoma cell lines, whose relation to ER^pos^ normal breast cells at best remains speculative.

Here we first identify the ER^pos^ cells *in situ* and search for markers that allow their subsequent tracking in culture. We then describe culture conditions for primary ER^pos^ cells in the presence of small-molecule inhibitors of transforming growth factor-beta (TGFβ) signalling. Importantly, these conditions also yield ER^pos^ cells from luminal ER^neg^ progenitors but not from basal cells. We envision that the present protocol will serve to bridge the existing gap of knowledge between normal human breast, which contain a small pool of ER^pos^ cells and the overwhelming ER expression found in the majority of breast cancers.

## Results

### Identification and isolation of normal ER^pos^ HBECs

**Figure 1:**
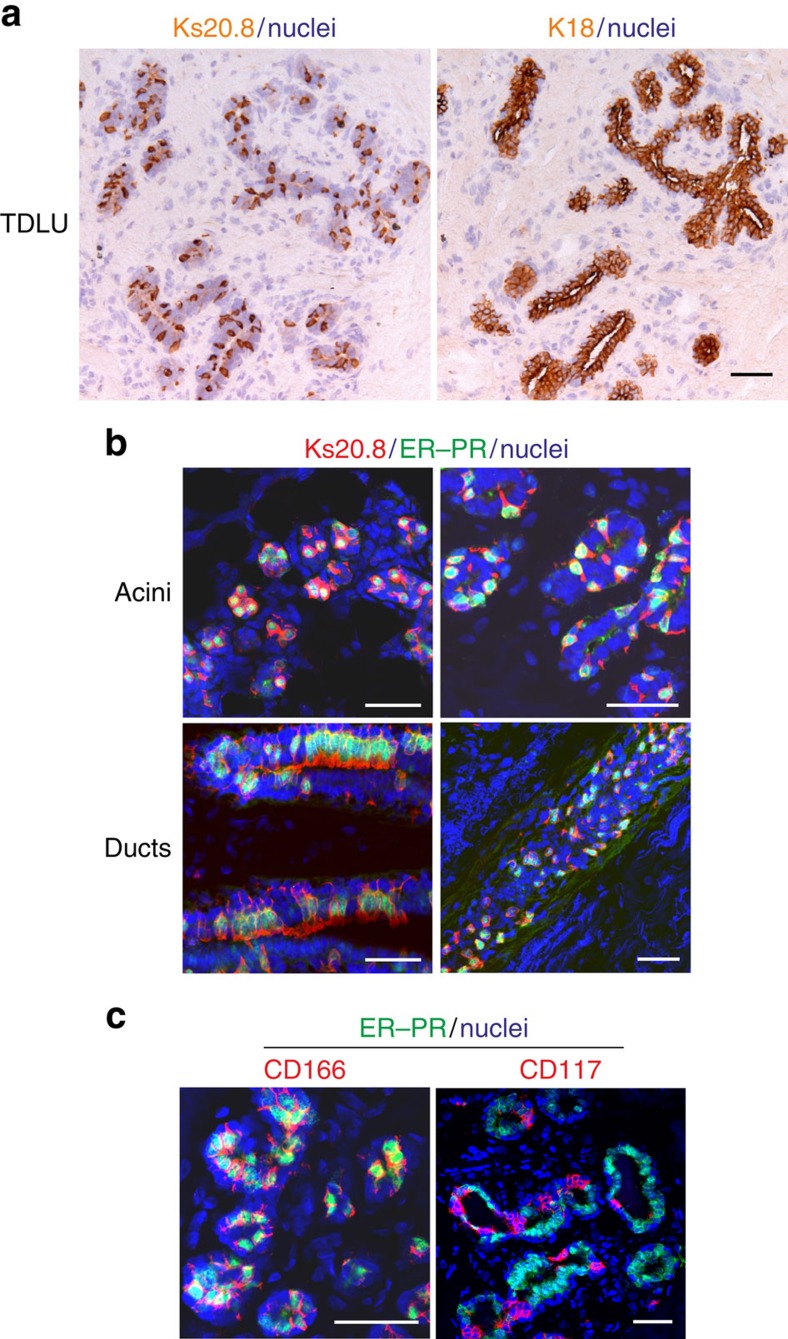
A unique staining signature Ks20.8^pos^/CD166^high^/CD117^low^ is eligible for ER^pos^ cell sorting and tracking. (**a**) Serial cryostat sections of a normal human breast terminal duct lobular unit (TDLU) stained with immunoperoxidase against Ks20.8 (left) and K18 (right), and counterstained with haematoxylin. Note the characteristic scattered staining pattern with Ks20.8 against the uniform lineage-related staining of luminal cells with K18. Scale bar, 50 μm. (**b**) Multicolour imaging of normal human breast cryostat sections including acini and ducts from four different biopsies stained for Ks20.8 (red), hormone receptors ER–PR (SP1–SP2, green) and DAPI nuclear stain (blue). The Ks20.8^pos^ compartment encompasses the ER–PR-expressing cells. Scale bar, 50 μm. (**c**) Multicolour imaging of normal human breast cryostat sections stained for CD166 (3A6, red), CD117 (K44.2, red), hormone receptors ER–PR (green) and DAPI stain (blue). While CD166 is highly expressed in hormone receptor-positive cells, CD117 shows the complementary pattern. Scale bar, 50 μm.

To answer the long-standing question of whether loss of hormone receptors in culture is due to the loss of cells or the loss of receptor protein expression, it was necessary first to provide tools for cell tracking and sorting of the relevant cells. To unequivocally track ER^pos^ HBECs at the single-cell level we screened our antibody library for surrogate markers with a long half-life, for example, cytokeratins^13^, in culture. *In situ* staining of more than 30 reduction mammoplasties revealed a surprising pattern with a monoclonal antibody (clone Ks.20.8) originally raised against cytokeratin 20, a simple epithelial cytokeratin with a very restricted expression pattern and not expressed in normal human breast^14^. The lack of true cytokeratin 20 expression in normal breast was here confirmed with two specific antibodies (listed in Table 1). Instead, Ks20.8 stained a subpopulation of luminal cells in a unique scattered pattern (Fig. 1a). Ks20.8 antibodies from four different suppliers (Table 1) revealed similar staining patterns (Supplementary Fig. 1a). The characteristic staining pattern led us to speculate that it indeed represented ER^pos^ cells. While ER and Ks20.8 apparently co-localized in acini as well as in ducts (Supplementary Fig. 1b), the immunofluorescence staining was not sufficiently strong to allow quantification. For this purpose, it was necessary to apply dual antibody immunofluorescence. Since ER and progesterone receptor (PR) are expressed coordinately in essentially the same cells in the normal human breast^15^, we decided to enhance immunofluorescence staining of uncultured cells by mixing antibodies for ER and PR. This approach revealed that the scattered staining with Ks20.8 encompassed almost the entire population of hormone receptor-positive HBECs in acini as well as in ducts (Fig. 1b) with the most evident co-localization in foci with the strongest receptor expression (in four out of eight biopsies tested). The antibody screen unravelled a number of additional markers of Ks20.8^pos^ HBECs some of which are well-known markers of ER^pos^ cells, including activating enhancer-binding protein 2 beta (AP2β), a marker of luminal differentiation, GATA3 (ref. 16), a marker of cell survival/longevity, Bcl2, two TGFβ-mediated, epithelial–mesenchymal transition-related markers, *N*-glycan/CDw75 antigen^17^ and *N*-cadherin (reviewed in ref. 18), as well as the stem cell markers, ALCAM (CD166) (ref. 19) and the laminin receptor, 67LR (ref. 20; Supplementary Fig. 2).

**Figure 2:**
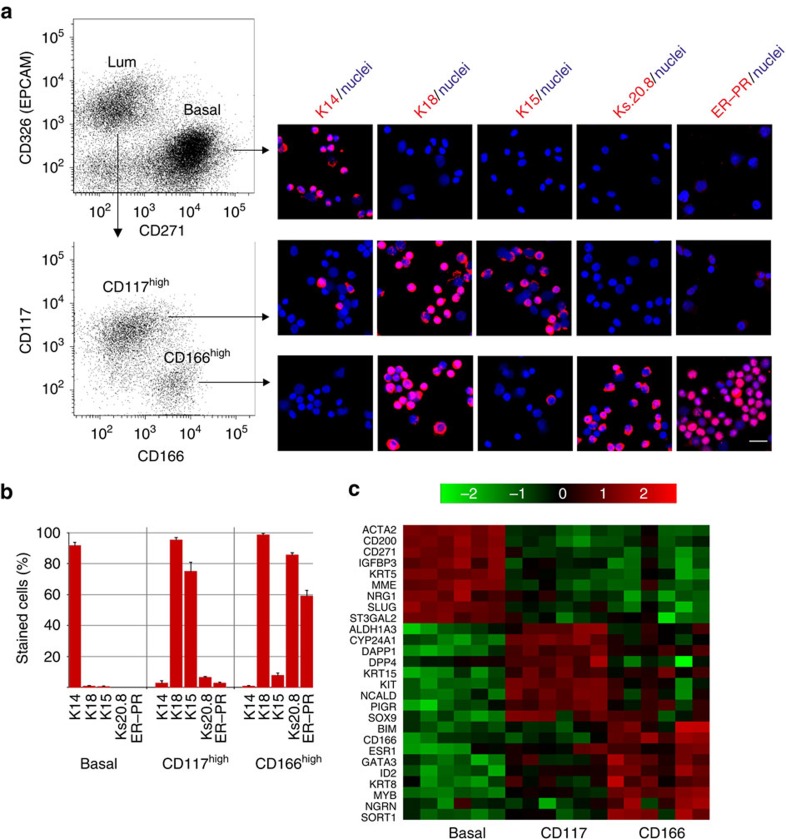
ER^pos^ cells are purified and tracked by sequential CD326/CD271–CD166/CD117 FACS followed by multicolour staining and qRT–PCR. (**a**) Multicolour flow cytometry of uncultured HBECs incubated with CD326/CD271/CD166/CD117 and visualized pairwise (left diagrams) to recover luminal cells (CD326^high^) and basal cells (CD271^high^) and from the luminal gate CD166^high^ and CD117^high^ cells. Smears of sorted cells were stained (right panel) with either of the markers against basal cells, cytokeratin K14; luminal cells, cytokeratin K18; luminal progenitors, cytokeratin K15; Ks20.8 or ER–PR and counterstained with DAPI nuclear stain. Hormone receptor-positive cells are observed primarily among CD166^high^ cells. Scale bar, 50 μm. (**b**) Purity of sorted cells as determined by staining of smears followed by quantification of the percentage of cells stained with either of the markers cytokeratin K14, K18, K15, Ks20.8 or hormone receptors (ER–PR; 3 × 100 cells per slide, error bars indicate s.d.'s). (**c**) Heatmap representing qRT–PCR analysis of the relative gene expression of lineage markers in sorted basal cells (basal), CD117^high^ luminal cells (CD117) and CD166^high^ luminal cells (CD166) from six different biopsies. Data confirm lineage-specific transcriptional profiles of the three cell populations and restricts ER expression (ESR1) primarily to CD166^high^ luminal cells. Colour bar indicates the fold difference of the relative gene expression in log_2_ scale.

For cell sorting purposes we found that CD166 and CD117 made good candidate surface markers of potential ER^pos^ and ER^neg^ cells, respectively—again as revealed by enhanced multicolour immunofluorescence (Fig. 1c). The mutual exclusivity of CD117 and ER (Fig. 1c) confirms what has been reported by others^21^. Accordingly, we designed a fluorescence-activated cell sorting (FACS) protocol to first separate the basal cell population from the luminal cell population based on EpCAM (CD326) and NGFR (CD271) followed by sorting with CD166 and CD117 to further dissect the luminal compartment (Fig. 2a and Supplementary Fig. 3). This protocol yields three populations, the purity of which was assessed by staining smears with lineage and progenitor markers K14, K18 and K15 (ref. 22), ER–PR as well as the novel ER^pos^ cell surrogate marker Ks20.8 (Fig. 2a). As expected, we found that ER^pos^/PR^pos^ HBECs were highly enriched in the CD166^high^/CD117^low^ gate (Fig. 2b). While immunofluorescence staining for ER alone evoked a cytoplasmic background staining, which prevented reliable quantification, the smears turned out to be ideal for accurate assessment of the level of co-localization of ER–PR and Ks20.8. Indeed, up to 90% of Ks20.8^pos^ cells were also ER–PR^pos^ (Supplementary Fig. 4) and up to 87% of ER–PR^pos^ cells were Ks20.8^pos^. The separation of the three subpopulations was further validated by quantitative reverse transcription–PCR (qRT–PCR), which confirmed a high ER expression (*ESR1*) in the CD166^high^ cells compared with the other subpopulations (Fig. 2c). An additional panel of markers further distinguished the two luminal subpopulations from the basal cell population (Supplementary Fig. 5). Importantly, we found that known ER signalling-related genes such as trefoil factor family-1 (TFF1; ref. 23) and growth regulation by oestrogen in breast cancer 1 (GREB1; ref. 24) were highly expressed in CD166^high^ cells as compared with other HBECs (Supplementary Fig. 5). The degree of separation in the CD166/CD117 FACS analysis was, however, somewhat biopsy dependent. In a series of six biopsies originating from women between 19 and 44 years old, five exhibited a similar separation with 11–49% of the cells being CD166^high^/CD117^low^, while one biopsy apparently did not contain a CD117^high^ population (Supplementary Fig. 6). As an alternative, CD117 could be replaced with the laminin receptor 67LR in the CD166 FACS to obtain enriched ER^pos^ HBECs (80% increase in Ks20.8-positive cells in the 67LR^high^ gate versus the 67LR^low^ gate; Supplementary Fig. 7). In conclusion, the combination of Ks20.8, CD166 and CD117 is a promising marker for ER^pos^ HBEC tracking and sorting.

### ER protein is lost in culture

**Figure 3:**
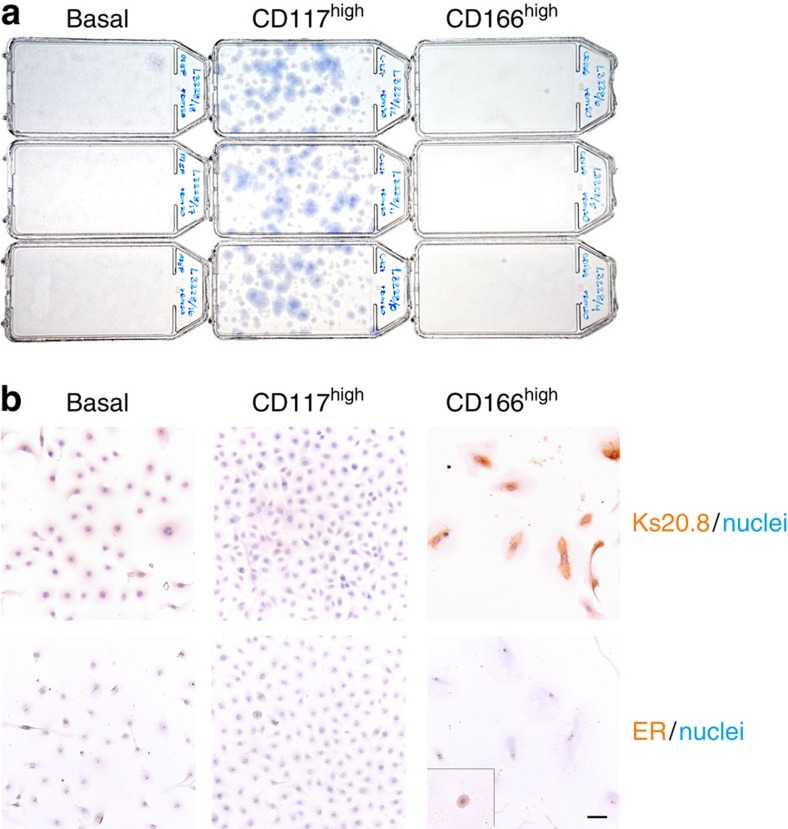
Loss of ER^pos^ cells in culture is due to both lack of growth and down modulation of ER expression. (**a**) T25 flasks in triplicate of basal cells (basal), CD117 ^high^ and CD166^high^ ER^pos^ luminal cells plated at a clonal density of 10^4^ cells per flask (400 cells per cm^2^) and stained with haematoxylin after 14 days in culture. Note that only CD117^high^ cells are colony forming at clonal density. (**b**) A higher magnification of cultures started at a higher cell density (3,000 cells per cm^2^) and immunoperoxidase stained at day 9 for Ks20.8 (upper row) or ER (SP1 prediluted, lower row; inset shows positive staining for ER at day 1 after plating) and counterstained with haematoxylin. Whereas ER expression is rapidly lost, the non-colony-forming cells of the CD166^high^ cultures remain Ks20.8 positive and traceable in culture. Scale bar, 50 μm.

With the surrogate markers in hand we were now able to examine the behaviour of sorted ER^pos^ HBECs in culture, potentially irrespective of ER expression. From the point of view that favourable conditions for luminal epithelial cells also apply to ER^pos^ HBECs, we refined a protocol to permit ample colony formation of luminal cells at clonal density. As cell culture plastic we used Primaria, a substrate with a high content of nitrogen previously shown to promote adhesion of breast cells^25^. The growth medium ‘FAD2' was modified from previously described media for culturing keratinocytes or basal breast epithelium on mouse fibroblast feeders^26^,^27^. The basal medium is Ham's F12:DMEM (1:3) similar to that of Tan *et al.*^27^, but with less serum, that is, 5%, as in Liu *et al.*^26^ Under these conditions, we gauged for colony formation among the three FACS gated populations described above. When plated at a clonal density of 400 cells per cm^2^, indeed colony forming luminal cells from the CD117^high^ gate were highly favoured over basal cells (Fig. 3a). However, it was also clear that the ER^pos^ HBECs from the CD166^high^ gate entirely failed to form colonies under similar conditions. However, a scrutiny of cultures at a higher magnification revealed that CD166^high^ cells in fact did not disappear from the culture. Rather, they plated, survived and also stained with Ks20.8 as a testimony of their original identity, but they entirely refrained from growth and rapidly lost the ER protein (Fig. 3b). By comparison, other HBEC culture media, that is, M87A (ref. 28), MEGM or WIT-P-NC did not support plating of CD166^high^ cells. Therefore, we conclude that failure of culturing ER^pos^ HBECs is caused by both lack of growth and loss of ER expression under conditions otherwise favouring propagation of luminal epithelial cells.

### TGFβ inhibitors induce ER expression and release ER^pos^ HBECs

**Figure 4:**
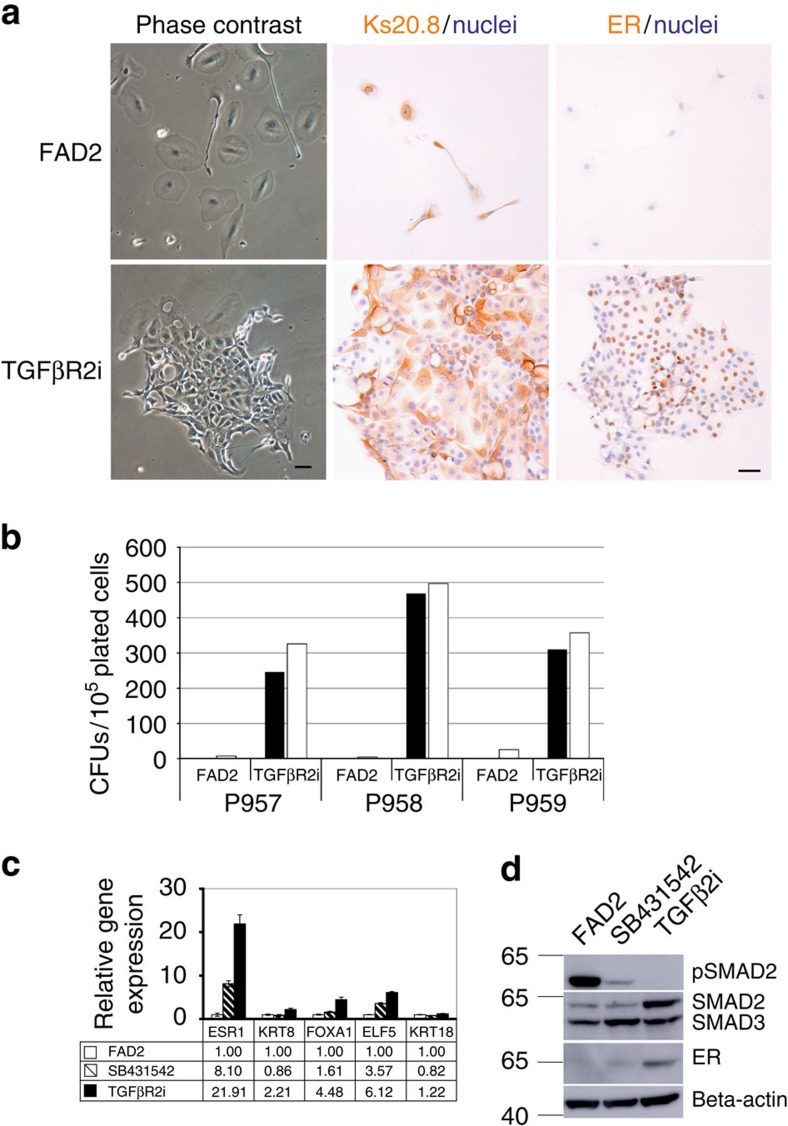
Relief of TGFβ-mediated negative regulation of growth releases ER^pos^ cells from quiescence. (**a**) Phase contrast micrographs (left column) after 8 days in culture and immunoperoxidase staining with Ks20.8 (middle column) and ER (SP1 prediluted, right column) of primary cultures of CD166^high^-derived cells in FAD2 (upper panel) or TGFβR2i (lower panel). Nuclei are counterstained with haematoxylin. Whereas Ks20.8^pos^ cells remain quiescent and ER negative on FAD2, they are colony forming and ER positive in TGFβR2i. Scale bar, 50 μm. (**b**) Quantification at day 13 of ER^pos^ (closed bar) or Ks20.8^pos^ (open bar) colony-forming units (CFUs) derived from CD166^high^/CD117^low^ cells from three consecutive biopsies (p957, p958 and p959) in FAD2 or TGFβR2i. In all three cases TGFβR2i supplied throughout 9–11 days supported colony formation of Ks20.8^pos^/ER^pos^ cells. (**c**) qRT-PCR of ER (ESR1), K8 (KRT8), FOXA1, ELF5 and K18 (KRT18) of RNA extracted from second-passage cells cultured for 5 days in FAD2 (open bars), in FAD2 with SB421543 (shaded bars) and in TGFβR2i (solid bars), respectively. Note the collective upregulation of ER and ER-associated gene expression in TGFβR2i. Error bars indicate s.d. of three technical triplicates. (**d**) Western blotting of proteins extracted at day 6 from second-passage CD166^high^/CD117^low^cells seeded at 4,000 cells per cm^2^ all cultured without EGF in FAD2 (left lane), in FAD2 with SB421543 (middle lane) and in TGFβR2i (right lane), respectively, incubated with antibodies recognizing phosphorylated SMAD2 (upper panel), SMAD2/3 (second panel), ER (third panel) and β-actin (lower panel). TGFβR2i inhibits pSMAD2 and upregulates ER protein expression.

We noted that earlier *in vivo* studies had implicated TGFβ1 signalling in the restraint of ER^pos^ mammary epithelial cells^29^,^30^. Here we therefore examined three small inhibitor molecules of TGFβ signalling, RepSox, SB431542 and SD208, and combinations hereof for their ability to relieve a potentially negative regulation of ER^pos^ HBEC growth in culture. We found that dual inhibition with SB431542 and RepSox, hereafter collectively termed TGFβR2i, recapitulated ER expression and stimulated ER^pos^ HBEC colony formation in four out of four biopsies. Moreover, the cells maintained Ks20.8 reactivity, albeit more widespread than ER expression compared with what is seen *in situ* (Fig. 4a). ER^pos^ cells from three different biopsies in low-density primary cultures in TGFβR2i (4,000 cells per cm^2^) exhibited a clonal capacity of 0.54%, 0.33% and 0.43%, respectively. By comparison, control conditions resulted in small, mostly abortive ER^neg^ clones (Fig. 4b). In general, ER expression was particularly evident in the dense centre of proliferating colonies or at confluency. Two different sources of SP1 antibody gave similar results (Table 1). Moreover, staining was confirmed with the less-sensitive ER antibody 1D5 (ref. 31). The response to TGFβR inhibitors was reproducibly observed in 10 out of 10 biopsies.

In addition to ER, TGFβR2i induced the expression of keratin K8 and the luminal cell transcription factors forkhead box protein A1, FOXA1 and E74-like factor 5, ELF5 (for reviews, see refs 32, 33) as revealed at the transcriptional level (Fig. 4c). This expression pattern was confirmed using another biopsy. TGFβR2i also upregulated the transcription of a number of genes known to be downstream targets of ER or modulators of ER activity, such as TFF1 (ref. 23) and insulin growth factor-binding protein 5 (IGFBP5; ref. 34; Supplementary Fig. 8a). Moreover, induction of ER protein expression to a significant degree was specific to RepSox as twice the concentration of SB431542 or replacement of RepSox with another ALK5 kinase inhibitor SD208 was insufficient to induce K8 and ER (data not shown). RepSox alone was not as effective in inducing ER or PR as in combination with SB431542 (Supplementary Fig. 8b). Likewise, while SB431542 alone was capable of inducing increased expression of ER at the mRNA level (Fig. 4c), this translated to the protein level to a significant degree only in the presence of RepSox (Fig. 4d and Supplementary Fig. 9). These findings suggest that ER expression is controlled through specific inhibition of TGFβ signalling. The presence of TGFβR in HBECs as well as the inhibition of phosphorylated SMAD2 concurrent with inhibitor-induced ER expression supported this (Supplementary Fig. 10 and Fig. 4d). Indeed, TGFβR2i culture was a key to sustained ER protein expression as removal of TGFβR2i led to complete loss of ER protein expression as observed in four independent experiments. Thus, in a representative experiment the frequency of ER^pos^ cells was reduced from 33.7% (s.d.±5.0%; 3 × 100 cells) in continuous TGFβR2i culture to 10.7% (s.d.±0.5%) after 3 days and to 0% after 5 days without TGFβR2i.

We next tested whether TGFβR2i would induce *de novo* ER expression in other subpopulations of luminal cells, for example, in the much more frequent CD117^high^-derived HBECs. Consistent with the presumed progenitor status of CD117^high^ cells in the human breast^35^, clones emerged from CD117^high^ cells that gained ER and stained positively for Ks20.8. This was, however, the only additional source we found of ER^pos^ HBECs, since TGFβR2i failed to induce ER in basal cells, in breast fibroblasts or in the established normal cell breast lines MCF10A or HMT-3522. Thus, both from preexisting ER^pos^ HBECs and from ER^neg^ progenitors, TGFβR2i readily provides growing colonies of ER^pos^ HBECs.

### TGFβR2i allows growth of ER^pos^ HBECs in serial subculture

**Figure 5:**
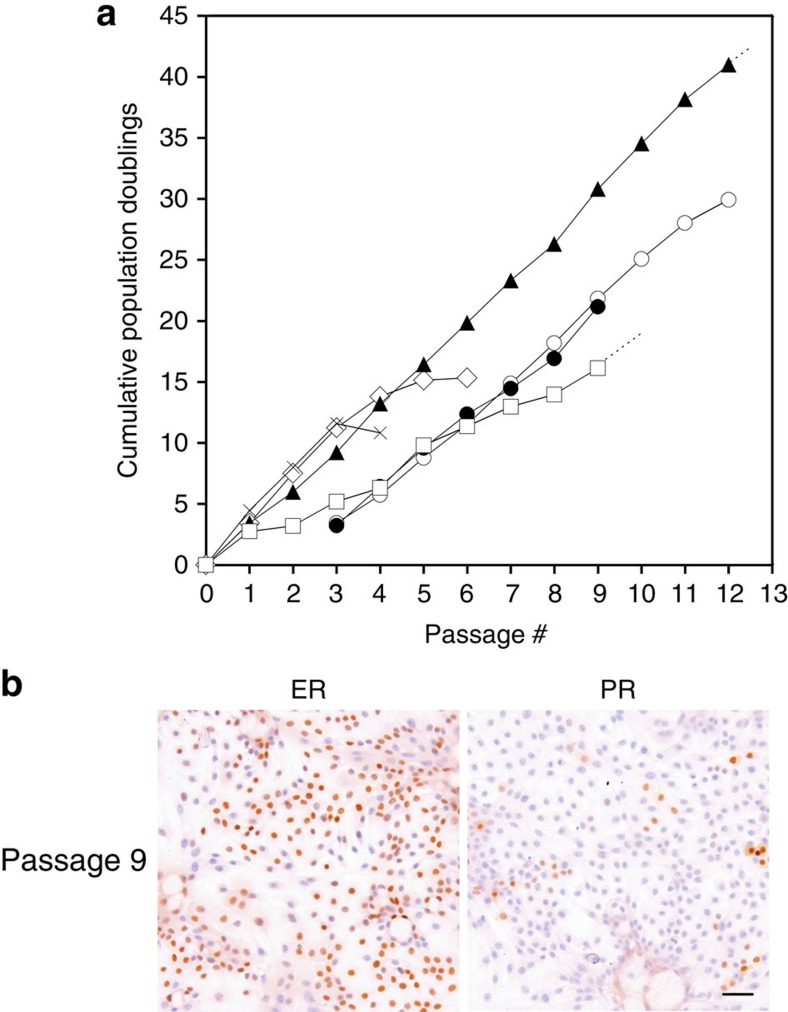
TGFβR2i allows efficient expansion of ER^pos^ cells. (**a**) Population doublings as a function of passage number calculated by continuous cell number recordings in triplicate cultures before confluency and plating at a fixed number of 4,000 cells per cm^2^ per flask at each split. TGFβR2i allows proliferation for up to six passages, corresponding to 15 population doublings (open diamond). If RepSox is omitted, the cells cannot be expanded beyond fourth passage (cross). Initial plating on 3T3 feeders with quantification starting in passage three extends proliferation to more than 10 passages, corresponding to more than 25 population doublings (open circle). Cells from a different sorting (albeit followed for a shorter period) exhibit similar extended proliferative capacity (closed circle). hTERT/shp16-transduced CD166^high^/CD117^low^ cells subsequently passaged at a fixed number of 6,000 cells per cm^2^ at each split extended the proliferative capacity even further (closed triangle), and the cells have now been growing for more than 12 passages. hTERT/shp16-transduced CD166^high^/CD117^low^ cells derived from a different biopsy, split at a ratio of up to 1:4, has so far been growing up to passage 9 (open square). (**b**) Even beyond 20 population doublings (passage 9), ER^pos^ cells with definitive lifespan maintain ER and PR expression as shown by immunoperoxidase and haematoxylin staining (cells in ninth passage seeded at 4,000 cells per cm^2^ and stained at day 5 with SP1 prediluted and SAN27, respectively). Scale bar, 50 μm.

TGFβR2i appeared to support growth of ER^pos^ HBECs also after passaging. To test this systematically, we plated CD166^high^-derived cells at a density of 6,400 cells per cm^2^ in primary culture, and the cultures were subsequently passaged at a density of 4,000 cells per cm^2^. On passaging, ER expression was particularly evident in the dense centre of proliferating colonies. Continuous proliferation under these conditions was maintained for up to six passages, corresponding to 15 population doublings (exemplified in Fig. 5a). Importantly, however, in the absence of RepSox, the cells could not be expanded beyond fourth passage (Fig. 5a). The lifespan and ER expression (ranging from 21 to 77% in second- to fourth-passage cultures, Table 2) were somewhat biopsy dependent, and in general proliferation slowed between fourth and sixth passages, and at the same time downregulation of ER expression was observed. While this narrowed the window of experimentation to up to fourth passage, the cells could easily and reproducibly be replaced with new cultures with similar luminal characteristics (Table 2). However, passage number could be increased by increasing seeding density. Thus, ER^pos^ cells derived from three biopsies could be cultured for another two to three passages, that is, up to passage six to nine when passaged at a high density of 8,000–20,000 cells per cm^2^. Moreover, the lifespan of ER^pos^ HBECs passaged at low seeding density could be further extended by initial plating on 3T3 feeders, which lead to proliferation for more than 10 passages, corresponding to more than 25 population doublings (Fig. 5a). Cells from a different sorting exhibited similar characteristics (Fig. 5a). We subsequently addressed whether TGFβR2i culture would allow for alternative approaches to extend the lifespan of normal breast-derived ER^pos^ cells. ER^pos^ HBECs were successfully transduced with pBABE-neo-hTERT and pLenti X2 hygro/shp16 constructs and have now been cultured for 4 months with weekly passages at 6,000 cells per cm^2^, exceeding 12 passages (Fig. 5a). Transduction of ER^pos^ cells from a different biopsy and subsequent splitting at up to 1:4 supported the robustness of this approach. Of note, these long-term cultured cells exhibited a phenotype essentially similar to cultures with definitive lifespan, including a relatively high level of ER expression (Supplementary Fig. 11 and Table 2). Thus, while the proliferation of ER^pos^ cells with definite lifespan is somewhat slow as it took more than 100 days to generate more than 25 population doublings, the present protocol nevertheless allows a considerable expansion of the ER^pos^ cell population extending from low-passage cultures with low seeding density through medium-passage cultures with high seeding density to high-passage cultures of hTERT/shp16-tranduced cells, which provides a relatively wide window of experimentation. Of note, until senescence the cells maintain their expression of ER, Ks20.8 reactivity as well as—mainly in densely packed colonies—expression of PR (Fig. 5b). In conclusion, TGFβR2i readily supports serial subculture of a population of ER^pos^ HBECs, that is, ER^pos^ progenitors.

### ER^pos^ progenitors respond to oestrogen

**Figure 6:**
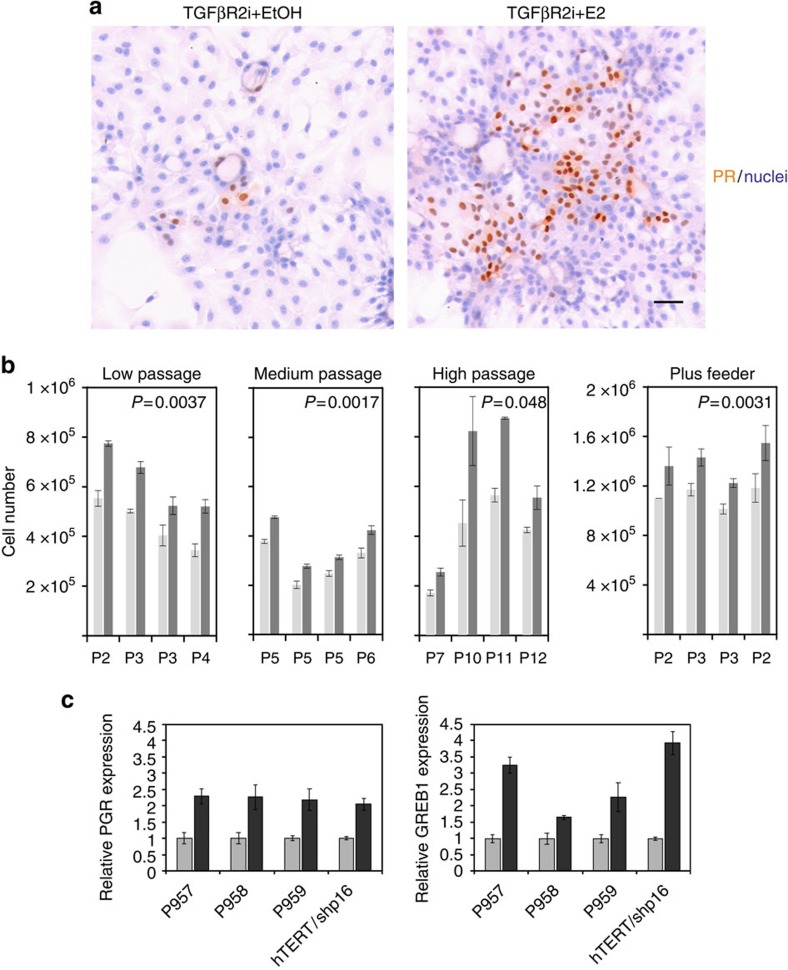
ER^pos^ cells in culture respond to oestrogen. (**a**) Staining of PR (SAN27) in second-passage CD166^high^ cells deprived of EGF and exposed to vehicle (EtOH) or oestrogen (E2) for 10 days. ER^pos^ cells respond to oestrogen by increased expression of PR. (**b**) Quantification of cell number in four sets of pairwise triplicate cultures of EGF-deprived, oestrogen-stimulated CD166^high^ low-passage cells grown for 4 days in second (P959) or third passage (P958), or 13 days in fourth passage (P959); medium-passage cells grown at high density before growth for 6 days (P958), 15 days (P957) and 14 days (P957 cultured without EGF since second passage) in fifth passage or cells with extended lifespan grown for 14 days in sixth passage; or high-passage cells (hTERT/shp16) in seventh passage grown for 13 days or in tenth to twelfth passage grown for 11, 15 and 11 days, respectively, in the presence of oestrogen (dark grey) or vehicle (light grey); or pairwise triplicate cultures of CD166^high^ cells plus feeder grown for 8 days in the presence of oestrogen (dark grey) or vehicle (light grey). Second-passage cultures derived from two different biopsies (first and fourth set of bars) do not respond differently from third-passage cultures (second set of bars), irrespective of omission of EGF from TGFβR2i before co-culture (second set of bars)). Omission of EGF throughout the entire experimental period reduced the total cell number, but did not augment the response to oestrogen (third set of bars). Error bars indicate s.d.'s. In all cases, oestrogen treatment significantly increases the growth of ER^pos^ cells, independent of passage group (by analysis of variance (ANOVA), *P*<0.05). The difference between experimental and control in each passage group is statistically significant by paired *t*-test (*P* values in each diagram). (**c**) Relative expression in triplicate of PGR and GREB1 from second (P957 and P959) and third passages (P958), or fourteenth passage long-term (hTERT/shp16) cultured ER^pos^ cells exposed to vehicle (EtOH, grey bars) or oestrogen (E2, black bars) for 4 (P958 and P959), 22 (P957) or 13 (hTERT/shp16) days, respectively. Transcription of downstream target genes of ER signalling, PGR and GREB1 is in all cases significantly upregulated by oestrogen as assessed by two-tailed *t*-test, *P*<0.05. Error bars indicate s.d.'s.

As an ultimate test for a physiologically relevant ER expression we decided to assess the effects of added cognate ligand, that is, oestrogen (β-oestradiol). Accordingly, ER^pos^ cells were exposed to 10^−8^ M of β-oestradiol or vehicle. The effect of oestrogen was tested in the absence of epidermal growth factor (EGF) since the transcriptional activation of ER can be induced by EGF-induced MAP kinase activity^36^. As a first indication of a functional ER, a higher focal expression of PR protein, a downstream target of ER signalling, was seen in the presence of oestrogen (Fig. 6a). Low-passage cultures (passage 2–4) were exposed to oestrogen or vehicle, and the cell number was quantified. As seen in Fig. 6b, significantly higher cell numbers were recorded in cultures with oestrogen (Fig. 6b). Medium-passage cultures (passage 5–6) reached by high seeding density exhibited a similar response, albeit in general exhibiting a slower growth (Fig. 6b). Likewise, a proliferative response was observed in high-passage culture of hTERT/shp16-transduced ER^pos^ cells (passage 7–12; Fig. 6b). That the observed increase in cell number was indeed due to the specific effect of signalling through ER was verified on addition of the ER antagonist ICI182,780, which could abolish the effect completely (Supplementary Fig. 12). Long-term exposure to oestrogen further augmented the oestrogen response at the transcriptional level, as indicated by increased expression of PGR and GREB1 (Fig. 6c), as well as at the translational level as observed in 10 preparations from three biopsies tested in passage 2–6 (Supplementary Fig. 13a) with a significant overlap in ER and PR protein expression (Supplementary Fig. 13b). Thus, the cultured ER^pos^ cells retain their ability to respond to oestrogen in a physiologically relevant manner. As an alternative approach we plated ER^pos^ cells on fibroblast feeders, which have been used previously to reveal an oestrogenic response in mouse mammary cells^37^. ER^pos^ cells were plated on confluent fibroblast feeders in TGFβR2i with or without oestrogen. At day 8, there was a significant increase in cell number with oestrogen as compared with the control (Fig. 6b). In the presence of fibroblasts, ER^pos^ cells responded to oestrogen irrespective of the presence of EGF (Fig. 6b). These data imply that the stromal microenvironment beyond the influence of TGFβ is important in the regulation of the growth of ER^pos^ cells.

In conclusion, we have developed a method to isolate, track and subculture ER^pos^ and oestrogen-responsive cells from normal human breast.

## Discussion

In spite of the fact that there are multiple protocols for enrichment, long-term cultivation and clonal growth of HBECs, none of them are able to isolate, track or support the growth of ER^pos^ HBEC. This led us to screen our antibody repository for antibodies against human breast antigens eligible for flow cytometry and cell tracking, and to test whether the reluctant growth of ER^pos^ HBEC in culture is due to inhibitory TGFβ signalling. Our present findings demonstrate that ER^pos^ HBECs identified *in situ* by a panel of markers, including PR, AP2β, GATA3, Bcl2, CDw75, *N*-cadherin and 67LR, are enriched for in a CD326^high^/CD271^low^/CD166^high^/CD117^low^ gate, and these cells express an antigen, unexpectedly decorated by an antibody, clone Ks20.8, originally raised against cytokeratin 20 (ref. 14). The distinction between ER^pos^ cells, other luminal cells and basal cells is further supported by their relative expression of a panel of transcripts.

The prospective isolation and tracking of ER^pos^ single cells from normal breast tissue hold promises for the future comparisons between normal, benign and malignant ER^pos^ cells, which will hopefully shed some light on the evolution and pathogenesis of the most frequent form of human breast cancer. Being able to isolate and track the cells, however, would be of limited value if the ER expression was lost on culture. It has been anticipated that ER^pos^ normal cells cannot divide and that this is why HBECs rapidly lose ER expression in culture^38^. We show here that ER^pos^ cells can be released from growth restraint and sustained by TGFβR2i and that ER^pos^ cells under these conditions can be expanded considerably. These findings may represent a paradigm shift in studying ER expression and function in the breast, in the future no longer relying exclusively on *in vivo* rodent models and human breast cancer cell lines. In addition, we believe the results answer the long-standing question of whether ER^pos^ cells can self-renew, and further establishes that ER^pos^ cells can be generated from ER^neg^ progenitors^39^,^40^,^41^,^42^, here represented by CD117^high^ luminal cells. Importantly, the response to TGFβR2i is specific to luminal cells only, as neither basal cells, fibroblasts nor ER^neg^ normal breast cell lines are able to switch on ER in response to TGFβR2i. The fact that ER^pos^ HBECs are here shown to be proliferating is in favour of the existence of ER^pos^ progenitors. In general, however, most ER^pos^ HBECs are considered to be close to the base in the hierarchy^35^. Thus, as has been hypothesized for breast cancer hierarchies^43^, our data could be interpreted in favour of the existence of bidirectionality also in the normal breast hierarchy, that is, on appropriate stimuli a subpopulation of apparently differentiated cells turn out in reality to be facultative progenitors.

The release of ER^pos^ HBECs in culture by TGFβR2i is clinically relevant because it may help explain an enigmatic difference between the normal human breast and breast cancers. Thus, in the normal breast *in vivo* there is a strict dissociation between steroid receptor expression and proliferation^15^. In breast cancer and to a varying degree in precancerous breast lesions this negative association is lost^44^. It has been speculated that this may represent an important early change in the genesis of breast cancer either reflecting a failure to downregulate ER as cells enter the cell cycle or a failure to suppress division of ER^pos^ cancer cells^44^. Our data indeed are in favour of the latter possibility because ER^pos^ HBECs do divide if TGFβ signalling is perturbed, a very likely scenario in cancer (for review see ref. 45). While in primary breast cancer a normal-like TGFβ signalling is still in operation to restrain growth, in metastatic breast cancer TGFβ signalling has shifted to that of an epithelial–mesenchymal transition response^45^.

Another implication of the TGFβR2i culture protocol is that it represents a much-in-demand cell-based assay for oestrogen action on normal cells. It is already well established that oestrogen is a mitogen for ER^pos^ breast cancer. However, its role in relation to ER^pos^ HBECs has remained a mystery due to lack of ER^pos^ cell culture models, and because ectopic expression of ER in basal cell lines under standard culture conditions has provided the paradoxical result of growth inhibition^11^,^12^. As a proof of principle, we here show that the TGFβR2i protocol serves as a physiologically relevant cell-based assay for oestrogen action, and moreover, in ER^pos^ cells, several oestrogen-responsive genes downstream of ER are upregulated. Thus, the TGFβR2i protocol represents a cell-based assay for gauging oestrogen action reminiscent of its action *in vivo*. Intriguingly, in the presence of fibroblasts, growth of ER^pos^ HBECs is stimulated by oestrogen irrespective of the presence of EGF. This implies that stromal factors modulate ER activity. With the relevant representatives of ER^pos^ cells in hand, the complexity of this interaction can now be elucidated.

Our data further suggest that TGFβ signalling is key to the CD166^high^ phenotype, and the exact mechanisms by which TGFβR2i generate ER^pos^ cells clearly await further scrutiny. It cannot be excluded, however, that what we observe is part of a more general association between CD166 expression and TGFβ signalling. Thus, very recently others have found that TGFβ signalling is a main driver of the behaviour of CD166-expressing prostate cells^46^.

The present protocol is the result of several years of experimentation including many reduction mammoplasties from different donors until the present conditions were established. We cannot exclude that further improvements are possible. The data illustrate, however, that apparent post-mitotic cells *in vivo*, given the right conditions may multiply considerably, yet still exhibiting a definitive lifespan, and the approach moreover serves as a platform for extending the lifespan of ER^pos^ cells into serial subculture.

While we have reproducibly recovered and propagated ER^pos^ cells from all biopsies tested, we nevertheless wish to emphasize on a number of technicalities, which should be carefully observed when the protocol is applied. Biopsies inherently vary with respect to the frequency of ER^pos^ cells^5^, which may eventually influence the definite number of ER^pos^ cells that can be derived from a given biopsy. Biopsies also vary with respect to growth potential, but in our hands they all perform well up to at least passage 4 (Table 2). It should be kept in mind, however, that the protocol releases only a minority of the total number of initially quiescent ER^pos^ cells, that is, up to 0.54% when plated at 4,000 cells per cm^2^ on sorting. Also, we strongly recommend that any attempts to validate Ks20.8 as well as co-expression of ER and PR by multicolour immunofluorescence are accompanied by immunoperoxidase staining to unravel the true overlap in staining. This is particularly important because we found that tracking of ER^pos^ HBECs in smears must rely on either the ER/PR antibody mixture or the Ks20.8 surrogate marker specifically combined with the ER fixation protocol. Last, we would like to draw the attention to the fact that while the cultured CD166^high^/CD117^low^ cells for the major part retain an elaborate profile of ER^pos^ cells *in situ,* they may—again somewhat dependent of the biopsy of origin—display focal traits of basal or myoepithelial cells, such as p63, concurrently with luminal traits (Table 2).

That said, there are multiple implications of the present protocol. First, knowledge about how to turn on and off the ER expression in non-malignant breast epithelial cells may offer an alternative to selective oestrogen receptor modulators in prevention of breast cancer in women with elevated risk of disease^47^. Second, a reproducible source of normal human ER^pos^ HBECs will represent a first step towards a physiological, cell-based screen for environmental oestrogenic activity and susceptibility of normal cells to breast cancer^48^. And third, the protocol may serve to test the functional role of recently identified single-nucleotide polymorphisms associated with increased risk of breast cancer^49^. In conclusion, we have provided a cell-based assay that allows normal breast ER^pos^ cells to present themselves for investigation. These findings may fuel future advances in breast cancer prevention.

## Methods

### Tissue

Normal breast biopsies were collected with consent from women undergoing reduction mammoplasty for cosmetic reasons. The use of human material has been reviewed by the Regional Scientific Ethical Committees (Region Hovedstaden) and approved with reference to H-2–2011–052. Normal breast tissue was prepared as described^25^. In brief, normal breast specimens were minced with opposing scalpels and treated overnight at 37 °C with rotation in collagenase (Type 3, Worthington Biochemical Corporation, 900 U ml^−1^ in DMEM/F-12 (Life Technologies, catalogue no. 21041) with 2 mM glutamine (Sigma, G7513) and 50 μg ml^−1^ gentamycin (Biological Industries)) before differential centrifugation to aspirate dissolved fat and to isolate epithelial organoids and fibroblasts. These were then either used directly or frozen in liquid nitrogen for later use.

### Fluorescence-activated cell sorting

To reveal epithelial cell composition and to isolate single cells, organoids from 13 biopsies were trypsinized, filtered through a 100-μm filter and resuspended in HEPES buffer supplemented with 0.5% BSA (bovine fraction V; Sigma-Aldrich) and 2 mM EDTA (Merck), pH 7.5. The suspended cells were incubated for 45 min at 4 °C in the presence of conjugated monoclonal antibodies EpCAM/CD326-PerCP cy5.5 (9C4, 1:20, BioLegend), NGFR (neurotrophin receptor, p75)/CD271-APC (ME20.4, 1:50, Cedarlane Laboratories) to separate basal and luminal cells, and activated leukocyte cell adhesion molecule CD antigen, ALCAM/CD166-AF488 (3A6, 1:20, AbD Serotec) and C-Kit/CD117-PE (104D2,1:20, BD) to separate luminal cells into luminal ER^neg^ (CD166^low^/CD117^high^) cells and luminal ER^pos^ (CD166^high^/CD117^low^) cells. On incubation, the cells were washed twice in HEPES/BSA/EDTA buffer and filtered through a 20-μm filter cup (Filcons) to prevent clogging of the FACS apparatus. Propidium iodide (Invitrogen) was added at a concentration of 1 μg ml^−1^, and the cells were analysed and sorted using a flow cytometer (FACSAria; BD Biosciences). An alternative way to isolate the two luminal subpopulations is to incubate for 30 min with EpCAM/CD326-PerCP cy5.5 and ALCAM/CD166-AF488 along with 67 kDa Laminin Receptor (MLuC5 1:50, Abcam) followed by 20 min incubation with secondary antibody Alexa Fluor 647 Goat Anti-Mouse IgM (1:500, Life Technologies).

To detect TGFβ receptors on the cell surface, third-passage CD166^high^/CD117^low^ cells were incubated with monoclonal antibody TGFβRII (MM0056–4F14, 1:20, Abcam) followed by secondary antibody AF488 (IgG1, 1:500) and analysed by FACS. Overlay histograms were produced using Flowing Software 2.5.1 (University of Turku, Finland).

To establish fibroblast feeders, fourth-passage fibroblasts were incubated as described above with monoclonal antibodies CD26 (202–36, 1:200, Abcam) and conjugated CD105-AF488 (1:25, AbD Serotec) followed by secondary antibody AF647 (IgG2b, 1:500) to isolate CD105^high^/CD26^low^ cells.

### Cell culture

On sorting, the primary cell populations were plated in Primaria T25 flasks (#3813, Becton Dickenson) in the presence of ‘FAD2' ((DMEM, high glucose, no calcium, Life Technologies):Ham's F12 Nutrient Mixture (F12, Life Technologies) 3:1 v/v) with 2 mM glutamine, 0.5 μg ml^−1^ hydrocortisone, 5 μg ml^−1^ insulin, 10 ng ml^−1^ cholera toxin (Sigma-Aldrich), 10 ng ml^−1^ epidermal growth factor (Peprotech), 1.8 × 10^–4^ M adenine, 10 μM Y-27632 (Y0503, Sigma-Aldrich or 1683, Axon Medchem) and 5% fetal bovine serum (Sigma-Aldrich), modified from refs 26, 27). On plating, which could take up to 2 days for the luminal ER^neg^ (CD166^low^/CD117^high^) cells and thus determined the time point for addition of TGFβR2i, a combination of the selective inhibitor of TGFβ type I receptor activin receptor-like kinase ALK5, ALK4 and ALK7, SB431542 (ref. 50) (10 μM, S4317, Sigma-Aldrich or 1661, Axon Medchem) and an inhibitor of autophosphorylation of ALK5, RepSox^51^,^52^ (25 or 50 μM, R0158, Sigma-Aldrich) was added. To test the specificity of the effect of TGFβR2i, in some experiments SB431542 alone or the double concentration of SB431542 was used instead of TGFβR2i, or RepSox was replaced by another TGFβ type I receptor activin receptor-like kinase ALK5 inhibitor, SD208 (1 μM, Tocris Bioscience). The vehicle, dimethyl sulfoxide (Sigma-Aldrich), was included in all experiments in appropriate concentrations for control cultures. Gentamycin (50 μg ml^−1^, Biological Industries) was added to the cultures throughout the first week after sorting, otherwise antibiotics were not included. To address whether propagation of ER^pos^ cells was restricted to the FAD2 medium, CD166^high^/CD117^low^ cells sorted from one of the biopsies were plated at a density of 4,000 cells per cm^2^ on Primaria six-well in either FAD2, M87A^28^, MEGM (LONZA) or WIT-P-NC (STEMGENT) medium including cholera toxin (100 ng ml^−1^) (ref. 53) and the cultures were observed for plating for 3 days. The possible induction of ER by TGFβR2i in other breast cells was further tested in MCF-10A^54^, HMT-3522 (ref. 55), fibroblasts purified from normal breast tissue^25^, CD117^high^ cells purified from three different biopsies, as well as in basal cells isolated by FACS as described above and cultured on irradiated NIH-3T3 feeders^26^ before exposure to TGFβR2i for up to 7 days followed by staining for ER and Ks.20.8. Fibroblasts were routinely grown to confluency on collagen-coated T25 flasks (Nunc, 8 μg collagen per cm^2^, PureColl, CellSystems) in DMEM/F12, with 2 mM glutamine and 5% FBS before co-culture with CD166^high^/CD117^low^ luminal cells. For phase contrast microscopy a Nikon Diaphot 300 microscope was used.

Cloning efficiency at low density in FAD2 was observed by plating the sorted basal cells, and the luminal populations CD166^low^/CD117^high^ and CD166^high^/CD117^low^, at 400 cells per cm^2^ and culturing for 14 days, followed by fixation in methanol for 5 min at −20 °C and counterstaining of nuclei with haematoxylin. To assess the Ks20.8 and ER expression of the sorted populations and to further assess whether starting the culture at a higher density would change the expression, cells were seeded at 3,000 cells per cm^2^ and cultured for 9 days before immunocytochemical staining.

To quantify the frequency of Ks20.8^high^/ER^pos^ colony-forming units CD166^high^/CD117^low^ luminal cells from three different biopsies were plated at a density of 4,000 cells per cm^2^ with or without TGFβR2i and grown for 13 days before staining for ER and Ks20.8 followed by quantification using an ocular grid. The number of stained colonies defined as presence of either marker in three areas of each culture relative to the initial number of seeded cells was calculated.

Low-density split cultures were initially seeded at 6,400 cells per cm^2^ and grown for up to 15 days in primary culture in TGFβR2i or with SB431542 alone. Next, the cultures were trypsinized and seeded at 4,000 cells per cm^2^ in triplicate cultures, and subsequently passaged at the same density before the cultures reached confluency. Parallel cultures were stained to assess Ks20.8, ER and PR expression status. The number of cells was quantified manually using a counting chamber. Medium-passage cultures were passaged at high seeding density, that is, 8,000–20,000 cells per cm^2^. For extended cultivation of low-density cultures sorted luminal Ks20.8^pos^/ER^pos^ (CD166^high^/CD117^low^) cells were first allowed to form colonies on irradiated NIH-3T3 feeders in modified breastoid base medium (BBM) without HEPES^56^ (DMEM/F12 supplemented with glutamine, 100 μM ethanolamine (Sigma , E0135), 1 μg ml^−1^ hydrocortisone (Sigma, H0888), 9 μg ml^−1^ insulin (Sigma, I6634), 5 μg ml^−1^ transferrin (Sigma, T1147), 5.2 ng ml^−1^ selenous acid (sodium salt, BD Biosciences, 354201), 20 ng ml^−1^ basic fibroblast growth factor (Peprotech, 100-18B) and 5nM amphiregulin (R&D Systems, 262-AR-100/CF)) with the addition of Y-27632, adenine and the serum replacement B27 (20 μl ml^−1^, Life Technologies), and subsequently sorted by FACS on incubation with CD326 and CD271 as described above to isolate CD326^high^/CD271^high^ cells before plating and passaging in TGFβR2i culture. Population doublings were calculated as *n*=3.32(log UCY−log *I*)+*X*, where *n*=population doubling, UCY=cell yield, *I*=inoculum number and *X*= population doubling rate of inoculum.

To determine whether continuous TGFβR2i culture was needed to sustain ER and Ks20.8 expression, cells in the sixth passage were switched to FAD2 and the number of ER^pos^ cells (*n*=3 × 100) was compared with parallel TGFβR2i cultures at day 3 and 5. Three similar experiments were performed with cloned hTERT/shp16-transduced cells (please see below).

As an alternative approach to extend the lifespan of Ks20.8^pos^/ER^pos^ cells, the method by Kiyono *et al.*^57^ was adapted by introducing human telomerase (hTERT) and shRNA p16 (shp16) to second-passage CD166^high^/CD117^low^cells. The viral constructs, pLENTi X2 Hygro/shp16 (w192–1, AddGene #22264, a gift from Eric Campeau) and pBABE-neo-hTERT^58^ (AddGene #1774, a gift from Robert Weinberg) were prepared as follows: lentiviral particles containing the shp16 construct were generated by transient co-transfection into HEK-293T cells using the calcium phosphate method with the vesicular stomatitis virus glycoprotein expressing construct pCMV–vesicular stomatitis virus glycoprotein as well as pol–gag construct pLP1 and rev construct pLP2. Retroviral hTERT particles were generated by transient co-transfection of the vector construct into a pantropic retroviral packaging cell line GP2–293 (Clonetech), which stably expresses the retroviral gag–pol genes. Medium was changed ∼16 h after transfection and viral media was collected ∼48 h later and stored at 4 °C for further purification. High-titre stocks of the virus were purified using a 20% sucrose gradient during ultracentrifugation with a Beckman SW32Ti rotor at 25,000 r.p.m. for 1.5 h. Purified viral solution was stored at −80 °C.

Before transduction, the cells were first treated with 200 mU ml^−1^ neuraminidase (N7885, Sigma) for 2 h at 4 °C, and then transduced using a high-viral titre^59^ containing the pBABE-neo-hTERT construct in TGFβR2i without FBS and incubated at 37 °C in 5% CO_2_ overnight. The culture medium was changed and the transduced cells underwent antibiotic selection for 10 days with 500 μg ml^−1^ G418 (Life Technologies). On confluency, the cells were passaged and underwent an identical transduction procedure with viral particles containing the pLenti X2 Hygro/shp16 construct and subsequent antibiotic selection with 100 μg ml^−1^ hygromycin (Sigma) for more than 2 weeks. The efficiency of the protocol was confirmed by hTERT/shp16 transducing second-passage CD166^high^/CD117^low^/Ks20.8^pos^/ER^pos^ cells derived from a different biopsy.

To test the proliferative response to oestrogen in low-passage (p2–4) cultures, ER^pos^ cells in TGFβR2i culture were seeded with 4,000 cells per cm^2^ in TGFβR2i with 25 μM RepSox and without EGF, supplemented with oestrogen (10^–8^ M, β-oestradiol, E2758, Sigma-Aldrich) and cultured for up to 13 days with medium change every other day. A primary culture from another biopsy was cultured 27 days in the presence of oestrogen and without EGF before splitting. The cells were passaged at 4,000 cells per cm^2^ in triplicate cultures with oestrogen or vehicle (96% ethanol). At day 4, the cultures were trypsinized and quantified using a counting chamber. Medium-passage (p5–6) cultures were passaged at 10,000–20,000 cells per cm^2^ before seeding at 4,000 cells per cm^2^, and the high-passage (p7–12) cultures including two hTERT/shp16-transduced populations derived from separate biopsies were passaged at 6,000 cells per cm^2^ or split at up to 1:4 before the growth experiment with a seeding density of 6,000 cells per cm^2^. The effect of an oestrogen antagonist (ICI182,780, Sigma-Aldrich), was tested in sixteenth passage, single-cell-cloned hTERT/shp16-transduced cells seeded at 4,000 cells per cm^2^ in TGFβR2i exposed to vehicle or oestrogen (10^–10^ M) and increasing concentrations of ICI182,780 (10^–10^, 10^–9^ and 10^–8^ M). At day 6, cultures were trypsinized and quantified in a cell counter (Roche Innovatis).

To assess whether the response of ER^pos^ cells to oestrogen was modulated by the presence of fibroblasts, second- or third-passage ER^pos^ cells, with or without the omission of EGF in the previous passage, were plated at a density of 5,600 cells per cm^2^ on confluent fibroblast feeders in triplicate culture. The following day, the culture medium was switched to TGFβR2i with oestrogen or vehicle. Under these conditions, fibroblasts did not grow. At day 8, the cultures were trypsinized and the total cell number was quantified. Whether a continuous lack of EGF influenced the response to oestrogen was tested in a similar set-up, where EGF was omitted from the medium during the entire experimental period.

The long-term effect of oestrogen on ER and PR expression in cultures without EGF was tested in 10 preparations of pairwise cultures in passage 2–6 derived from three different biopsies. The cultures were grown up to 26 days with oestrogen and vehicle, respectively, and subsequently stained by immunoperoxidase or immunofluorescence (see below).

### RNA extraction and qRT–PCR

Total RNAs from sorted normal primary cells from eight biopsies were extracted using Trizol (Invitrogen) and were reverse transcribed using the High Capacity RNA-to-cDNA Kit (Applied Biosystems). For general gene expression profiling, 2 ng of total cDNA was used to perform quantitative real-time PCR using the TaqMan Gene Expression Assays (Applied Biosystems) on CFX384 Touch Real-Time PCR Detection System (Bio-Rad). The primers are listed in Supplementary Table 1, and real-time PCR conditions were the following: 50 °C for 2 min and 95 °C for 10 min, followed by 40 cycles at 95 °C for 10 s and 60 °C for 30 s. To quantify cDNA concentration represented by cycle threshold (Ct) values, Cts were determined at the initial period of exponential amplification in triplicates and the different PCR runs were adjusted by inter-run calibrators (Bio-Rad CFX manager 3.0). Each gene expression level was to the mean of four reference gene expressions (GAPDH, HPRT1, TBP, and TFRC). The qRT–PCR data were then visualized as a heatmap using the statistical programme R (ref. 60) or as a bar graph using the values calculated by the 2^−ΔΔCt^ method^61^. For qRT-PCR of ER, K8, FOXA1, ELF5 and K18, second-passage cultures of CD166^high^ cells at a density of 8,000 cells per cm^2^ in FAD2, SB431542 or SB431542 switched to TGFβR2i culture conditions for 5 days before RNA extraction were used.

The expression of ER signalling and its downstream target genes were analysed in CD166^high^ cells with or without TGFβR2i by using a RT^2^ Profiler PCR array (human oestrogen signalling, Qiagen) according to the manufacturer's instructions. Total 4 μg of RNA was used per array, which was performed in duplicates from two different biopsies. Three sets of E2- or ethanol (vehicle)-treated second- or third-passage ER^pos^ cells or hTERT/shp16-transduced cells were further analysed in triplicate for PGR and GREB1 expression with the TaqMan Gene Expression Assays in the same condition mentioned above, using 20 ng of cDNA in each PCR reaction.

### Western blotting

Whole-cell lysates were prepared for extraction by incubation in RIPA lysis buffer for 30 min at 4 °C with protease inhibitor cocktail (P8340, Sigma) and phosphatase inhibitor cocktail (Sigma, P5726). Proteins were separated by a 4–12% Novex Bis-Tris pre-cast polyacrylamide gradient gel (Life Technologies) and transferred to a polyvinylidene difluoride membrane using iBlot dry blotting system (Life Technologies). Molecular weight was indicated by using a pre-stained protein ladder (SM0671, Fermentas). After blocking 1 h in room temperature, the membrane was incubated with primary antibodies recognizing Smad2/3 (1:1,000, #3102, Cell Signaling), pSmad 2 (1:1,000, #3101, Cell Signaling), β-actin (1:5,000, A-5441, Sigma) or ER (1:500, NCL-ER-6F11/2, Novocastra) in blocking solution with gentle rocking overnight at 4 °C. Secondary antibodies conjugated with horseradish peroxidase (Dako) were incubated for 1 h at room temperature. Western blots were visualized using enhanced chemiluminescence solution (PerceECL 32106, Thermo Scientific) and a chemiluminescence imager (Amersham Image 600, GE Healthcare life sciences).

### Immunohistochemistry and cytochemistry

Cryostat sections, smears of sorted cells and monolayer cultures were prepared and stained by immunoperoxidase or immunofluorescence, including negative controls without primary antibodies^3^,^22^,^62^. Specifically, cellular smears for ER–PR staining were fixed in 4% paraformaldehyde (Electron Microscopy Sciences) followed by fixation in ice-cold methanol:acetone (1:1) for 10 min at −20 °C. Smears were then washed two times for 3 min and blocked for 10 min in 0.5% saponin (Sigma)/10% goat serum in PBS. All subsequent washing and incubation steps were performed in 0.5% saponin/10% goat serum. To visualize hormone receptor expression smears were incubated with anti-ER (SP1, 1:10) and anti-PR (SP2, 1:10) for 2 h at room temperature followed by 30 min with Alexa Fluor 568 Goat anti-Rabbit IgG (1:500, Invitrogen). For quantification of ER–PR and Ks20.8 expression, smears were fixed as described above and subsequently incubated with anti-ER (SP1, 1:10), anti-PR (SP2, 1:10) and Ks20.8 (1:10) for 2 h at room temperature followed by incubation for 30 min with Alexa Fluor 568 Goat anti-Rabbit IgG (1:500, Invitrogen) and Alexa Fluor 488 Goat anti-Mouse IgG2a (1:500, Invitrogen). Slides were mounted with ProLong Gold antifade reagent with 4,6-diamidino-2-phenylindole (DAPI; Life Technologies), and quantification of stained smears was routinely based on 3 × 100 cells, directly observed or evaluated in random micrographs obtained with a × 20 objective in the confocal microscope.

Antibodies are listed in Table 1. Many antibodies stain independently of fixation procedure, but of note, to stain for ER and PR, cultures were rinsed in PBS, pH 7.4, or sections were air dried before fixation, for 5 min at room temperature, in 3.7% formaldehyde, two rinses in PBS, fixation in methanol:acetone (1:1) for 5 min at  20 °C, two rinses in PBS, permeabilization in 0.1% Triton X-100 in PBS, twice for 7 min, rinse in PBS and kept wet before application of UltraV Block (Thermo Scientific). ER and PR expression in cultures with long-term oestrogen exposure was assessed by immunoperoxidase for ER (SP1, prediluted) and PR (SAN27 or PgR636) or by double-labelling immunofluorescence for ER (SP1, 1:10) and PR (PgR636, 1:10) with Alexa Fluor 488 Goat anti-Rabbit IgG (1:500) and Alexa Fluor 568 Goat anti-Mouse IgG1 (1:500) as secondary antibodies.

To stain for K15, UltraV Block was substituted for 10% normal goat serum in PBS. Immunofluorescence and peroxidase stainings were evaluated, quantified and photographed using a laser-scanning microscope (LSM 510 or LSM700; Carl Zeiss MicroImaging, Inc.) and bright-field microscopes (Laborlux S or DM5500B, Leica), respectively. For quantification of immunoperoxidase staining of ER, K8, K19 and p63, nuclei were counterstained with haematoxylin and counted in randomly selected fields using an ocular grid and given as the percentage of stained cells of a total of 1,000 cells evaluated with a × 25 objective.

## Additional information

**How to cite this article:** Fridriksdottir, A. J. *et al.* Propagation of oestrogen receptor-positive and oestrogen-responsive normal human breast cells in culture. *Nat. Commun.* 6:8786 doi: 10.1038/ncomms9786 (2015).

## Supplementary Material

Supplementary Information Supplementary Figures 1-13 and Supplementary Table 1

## Figures and Tables

**Table 1:** List of antibodies used for immunostaining and/or FACS analysis.

**Antibody**	**Clone**	**Company/Catalogue No.**	**Peroxidase**	**Fluorescence**	**FACS**
AP2β	—	Santa Cruz, sc-8976	—	1:50	—
BCL2	124	Dako, M0887	—	1:25	—
CDw75	LN1	NeoMarkers, MS-130-P	—	1:10	—
CD117	104D2	Dako, M7140	—	1:50	—
CD117	K44.2	Sigma, K0131	—	1:50	—
CD117, PE	104D2	BD Biosciences, 332785	—	—	1:20
CD166	3A6	BioLegend, 343902	—	1:50	—
CD166, Alexa Fluor 488	3A6	AbD Serotec, MCA1926A488	—	—	1:20
CD271, APC	ME20.4	Cedarlane, CL10013APC	—	—	1:50
CD326, PerCP/Cy5.5	9C4	BioLegend, 324214	—	—	1:10–1:20
TGFβRII	MM0056–4F14	Abcam, ab78419	—	—	1:20
ERα	SP1	Labvision, RM-9101-S	1:25	1:10	—
ERα	SP1	Labvision, RM-9101-R7	Prediluted, ready to use	—	—
ERα	1D5	Dako, M7047	1:100	1:25	—
GATA3	HG3–31	Santa Cruz, sc-268	—	1:25	—
Keratin 8	M20	Abcam, ab9023	1:50	1:50	—
Keratin 8	TS1	Novocastra, NCL-CK8-TS1	1:50	—	—
Keratin 20*	Ks20.8	Dako, M7019	1:25	1:10	—
Keratin 20*	Ks20.8	Dako, IR777	Prediluted, ready to use	—	—
Keratin 20*	Ks20.8	Abcam, ab854	1:10	—	—
Keratin 20*	Ks20.8	Genemed, 61–0018–2	1:25	—	—
Keratin 20*	Ks20.8	Santa Cruz, SC-52320	1:25	—	—
Keratin 20	IT-Ks20.10	Progen Biotechnik, 61054	1:25	—	—
Keratin 20	CK205	Novocastra, NCL-CK20–543	1:50	—	—
Keratin 8/18	NCL-5D3	Abcam, ab90102	—	1:50	—
Keratin 14	LL002	NeoMarkers, MS-115-P	1:300	1:25	—
Keratin 15	LHK15	NeoMarkers, MS-1068-P	—	1:25	—
Keratin 18	M9	Monosan, MON3006	1:100	—	—
N-cadherin	32	BD Transduction Laboratories, 610920	—	1:25	—
PR	SAN27	Vector Laboratories, VP-P987	1:100	1:25	—
PR	SP2	Labvision, RM-9102-S	—	1:10	—
PR	PgR 636	Dako, M356901–2	1:50	1:10	
67-kDa laminin receptor	MLuC5	Abcam, ab3099	—	1:50	1:50

^*^Originally described to recognize cytokeratin 20, but keratin 20 is not expressed in normal human breast^14^.

**Table 2:** Frequency of ER^pos^ and lineage marker-positive cells as a function of passage number in culture.

**Passage #**	**Per cent ER**^**+**^ **cells**	**Per cent K8**^**+**^ **cells**	**Per cent K19**^**+**^ **cells**	**Per cent P63**^**+**^ **cells**
2	41/21/24/38/42	100/100/100/ND/100	44/47/100/ND/60	0*/5/0/ND/0*
3	**76**†/47/22/50‡/26	**100**†/100/100/100‡/100	**100**†/41/62/100‡/76	**0**†/2/0/0‡/0
4	**77**†/**26**†/28/51†/23	**100**†/**100**†/100/100†/100	**100**†/**79**†/34/100†/76	**0**†/**0**†/0/0†/0
5	**29**†/**72**†/**39**/*38*/70/37	**100**†/**100**†/**100**/*ND*/ND/100	**79**†/**100**†/**48**/*ND*/29/99	**0**†/0*†/0*/*ND*/0*/6
6	**66**†/**27**/*42*/*22*	**100**†/**100**/*ND*/*97*	**100**†/**60**/*ND*/*69*	**0**†/0*/*ND*/*8*
7	**54**†/**28**/*30*/*25*/24/45†	**100**†/**100**/*ND*/*100*/96/ND†	**100**†/**69**/*ND*/*63*/94/ND†	**0**†/0*/*ND*/*3*/6/10†
8	**57**†/**22**/*34*/*18*/45†	**100**†/**100**/*100*/*100*/ND†	**100**†/**78**/*100*/*55*/85†	**0**†/**0**/*0*/*0*/12†
9	**52**†/**19**/*28*/87	**100**†/**100**/*100*/100§	**100**†/**100**/*100*/14	**0**†/**0**/*0*/0
10	*19*	*ND*	*ND*	*ND*

ND, not done.

The numbers in black (roman font; not bold, italic or underlined entries) indicate low-density split, ∼4,000 cells per cm^2^.

Bold entries indicate high-density split, ∼8,000–20,000 cells per cm^2^.

Italic entries denote cells with extended lifespan on initial cultivation in BBMYAB and mouse feeders.

The entries that are underlined denote hTERT/shp16-transduced cells.

The percentage of ER^pos^ and lineage marker-positive cells evaluated after immunoperoxidase staining of cultures and counterstained with haematoxylin. Aside from staining for ER (SP1, prediluted), the lineage markers included luminal keratin K8 (stained by M20 or TS1) and keratin K19, and myoepithelial P63. Each number refers to the frequency of stained cells among a total of 1,000 cells in randomly selected fields in a culture representing one biopsy. Cells were counted by use of a × 25 objective and a × 10 ocular equipped with a grid. Note that cultures remain essentially ER^pos^ and luminal like. At the onset of senescence (starting at passage 4 in low-density split cultures and up to passage 6–9 in high-density split cultures) there is a tendency for the ER staining to fade out. High-density cultures from two biopsies maintained a high ER expression on omission of EGF and addition of E2.

^*^Very few, small foci or individual cells.

^†^EGF omitted, E2 added.

^‡^+E2 in passage 1.

^§^Stained in passage 11.

## References

[b1] Petersen O. W. & Polyak K. Stem cells in the human breast. Cold Spring Harb. Perspect. Biol. 2, 1–15 (2010). 10.1101/cshperspect.a003160 PMC2857168 20452965

[b2] Hammond S. L., Ham R. G. & Stampfer M. R. Serum-free growth of human mammary epithelial cells: rapid clonal growth in defined medium and extended serial passage with pituitary extract. Proc. Natl Acad. Sci. USA 81, 5435–5439 (1984). 6591199 10.1073/pnas.81.17.5435 PMC391719

[b3] Petersen O. W. & van Deurs B. Growth factor control of myoepithelial-cell differentiation in cultures of human mammary gland. Differentiation 39, 197–215 (1988). 2468550 10.1111/j.1432-0436.1988.tb00094.x

[b4] Taylor-Papadimitriou J. *et al.* Keratin expression in human mammary epithelial cells cultured from normal and malignant tissue: relation to in vivo phenotypes and influence of medium. J. Cell. Sci. 94, 403–413 (1989). 2483723 10.1242/jcs.94.3.403

[b5] Petersen O. W., Høyer P. E. & van Deurs B. Frequency and distribution of estrogen receptor-positive cells in normal, nonlactating human breast tissue. Cancer Res. 47, 5748–5751 (1987). 3664479

[b6] Rønnov-Jessen L., Petersen O. W. & Bissell M. J. Cellular changes involved in conversion of normal to malignant breast: the importance of the stromal reaction. Physiol. Rev. 76, 69–125 (1996). 8592733 10.1152/physrev.1996.76.1.69

[b7] Kang K.-S. *et al.* Expression of estrogen receptors in a normal human breast epithelial cell type with luminal and stem cell characteristics and its neoplastically transformed cell lines. Carcinogenesis 18, 251–257 (1997). 9054615 10.1093/carcin/18.2.251

[b8] Graham J. D. *et al.* DNA replication licensing and progenitor numbers are increased by progesterone in normal human breast. Endocrinology 150, 3318–3326 (2009). 19342456 10.1210/en.2008-1630 PMC2703536

[b9] Tanos T. *et al.* Progesterone/RANKL is a major regulatory axis in the human breast. Sci. Transl. Med. 5, 1–10 (2013). 10.1126/scitranslmed.3005654 23616122

[b10] Stampfer M. R. & Bartley J. C. Induction of transformation and continuous cell lines from normal human mammary epithelial cells after exposure to benzo(a)pyrene. Proc. Natl Acad. Sci. USA 82, 2394–2398 (1985). 3857588 10.1073/pnas.82.8.2394 PMC397564

[b11] Lundholt B. K., Briand P. & Lykkesfeldt A. E. Growth inhibition and growth stimulation by estradiol of estrogen receptor transfected human breast epithelial cell lines involve different pathways. Breast Cancer Res. Treat. 67, 199–214 (2001). 11561766 10.1023/a:1017977406429

[b12] Zajchowski D. A., Sager R. & Webster L. Estrogen inhibits the growth of estrogen receptor-negative, but not estrogen receptor-positive, human mammary epithelial cells expressing a recombinant estrogen receptor. Cancer Res. 53, 5004–5011 (1993). 8402691

[b13] Denk H., Lackinger E., Zatloukal K. & Franke W. W. Turnover of cytokeratin polypeptides in mouse hepatocyes. Exp. Cell Res. 173, 137–143 (1987). 2445591 10.1016/0014-4827(87)90339-9

[b14] Moll R., Löwe A., Laufer J. & Franke W. W. Cytokeratin 20 in human carcinomas. A new histodiagnostic marker detected by monoclonal antibodies. Am. J. Pathol. 140, 427–447 (1992). 1371204 PMC1886432

[b15] Clarke R. B., Howell A., Potten C. S. & Anderson E. Dissociation between steroid receptor expression and cell proliferation in the human breast. Cancer Res. 57, 4987–4991 (1997). 9371488

[b16] Asselin-Labat M. L. *et al.* Gata-3 is an essential regulator of mammary-gland morphogenesis and luminal-cell differentiation. Nat. Cell Biol. 9, 201–209 (2007). 17187062 10.1038/ncb1530

[b17] Lu J. *et al.* β-Galactoside α2,6 sialyltransferase 1 promotes transforming growth factor-β-mediated epithelial-mesenchymal transition. J. Biol. Chem. 289, 34627–34641 (2014). 25344606 10.1074/jbc.M114.593392 PMC4263869

[b18] Wheelock M. J., Shintani Y., Maeda M., Fukumoto Y. & Johnson K. R. Cadherin switching. J. Cell Sci. 121, 727–735 (2008). 18322269 10.1242/jcs.000455

[b19] Levin T. G. *et al.* Characterization of the intestinal cancer stem cell marker CD166 in the human and mouse gastrointestinal tract. Gastroenterology 139, 2072–2082 (2010). 20826154 10.1053/j.gastro.2010.08.053 PMC2997177

[b20] Selleri C. *et al.* The metastasis-associated 67-kDa laminin receptor is involved in G-CSF-induced hematopoietic stem cell mobilization. Blood 108, 2476–2484 (2006). 16788104 10.1182/blood-2005-11-012625

[b21] Westbury C. B. *et al.* Genome-wide transcriptomic profiling of microdissected human breast tissue reveals differential expression of KIT (c-Kit, CD117) and oestrogen receptor-alpha (ERalpha) in response to therapeutic radiation. J. Pathol. 219, 131–140 (2009). 19562735 10.1002/path.2581

[b22] Villadsen R. *et al.* Evidence of a stem cell hierarchy in the adult human breast. J. Cell Biol. 177, 87–101 (2007). 17420292 10.1083/jcb.200611114 PMC2064114

[b23] Rio M. C. & Chambon P. The pS2 gene, mRNA, and protein: a potential marker for human breast cancer. Cancer Cells 2, 269–274 (1990). 2223388

[b24] Ghosh M. G., Thompson D. A. & Weigel R. J. PDZK1 and GREB1 are estrogen-regulated genes expressed in hormone-responsive breast cancer. Cancer Res. 60, 6367–6375 (2000). 11103799

[b25] Rønnov-Jessen L. & Petersen O. W. Induction of α-smooth muscle actin by transforming growth factor-β1 in quiescent human breast gland fibroblasts. Implications for myofibroblast generation in breast neoplasia. Lab. Invest. 68, 696–707 (1993). 8515656

[b26] Liu X. *et al.* ROCK inhibitor and feeder cells induce the conditional reprogramming of epithelial cells. Am. J. Pathol. 180, 599–607 (2012). 22189618 10.1016/j.ajpath.2011.10.036 PMC3349876

[b27] Tan D. W. M. *et al.* Single-cell gene expression profiling reveals funcional heterogeneity of undifferentiated human epidermal cells. Development 140, 1433–1444 (2013). 23482486 10.1242/dev.087551 PMC3596987

[b28] Garbe J. C. *et al.* Molecular distinctions between stasis and telomere attrition senescence barriers shown by long-term culture of normal human mammary epithelial cells. Cancer Res. 69, 7557–7568 (2009). 19773443 10.1158/0008-5472.CAN-09-0270 PMC2782785

[b29] Ewan K. B. R. *et al.* Proliferation of estrogen receptor-α-positive mammary epithelial cells is restrained by transforming growth factor-β1 in adult mice. Am. J. Pathol. 167, 409–417 (2005). 16049327 10.1016/s0002-9440(10)62985-9 PMC1603552

[b30] Choudhury S. *et al.* Molecular profiling of human mammary gland links breast cancer risk to a p27(+) cell population with progenitor characteristics. Cell Stem Cell 13, 117–130 (2013). 23770079 10.1016/j.stem.2013.05.004 PMC3703476

[b31] Bogina G. *et al.* Comparison of anti-estrogen receptor antibodies SP1, 6F11, and 1D5 in breast cancer: lower 1D5 sensitivity but questionable clinical implications. Am. J. Clin. Pathol. 138, 697–702 (2012). 23086770 10.1309/AJCPLX0QJROV2IJG

[b32] Augello M. A., Hickey T. E. & Knudsen K. E. FOXA1: master of steroid receptor function in cancer. EMBO J. 30, 3885–3894 (2011). 21934649 10.1038/emboj.2011.340 PMC3209791

[b33] Oakes S. R., Hilton H. N. & Ormandy C. J. The alveolar switch: coordinating the proliferative cues and cell fate decisions that drive the formation of lobuloalveoli from ductal epithelium. Breast Cancer Res. 8, 207–216 (2006). 16677418 10.1186/bcr1411 PMC1557712

[b34] McGuire W. L. Jr *et al.* Regulation of insulin-like growth factor-binding protein (IGFBP) expression by breast cancer cells: use of IGFBP-1 as an inhibitor of insulin-like growth factor action. J. Natl Cancer Inst. 84, 1336–1341 (1992). 1379645 10.1093/jnci/84.17.1336

[b35] Lim E. *et al.* Aberrant luminal progenitors as the candidate target population for basal tumor development in *BRCA1* mutation carriers. Nat. Med. 15, 907–915 (2009). 19648928 10.1038/nm.2000

[b36] Kato S. *et al.* Activation of the estrogen receptor through phosphorylation by mitogen-activated protein kinase. Science 270, 1491–1494 (1995). 7491495 10.1126/science.270.5241.1491

[b37] Haslam S. Z. Mammary fibroblast influence on normal mouse mammary epithelial cell responses to estrogen *in vitro*. Cancer Res. 46, 310–316 (1986). 3940197

[b38] Duss S. *et al.* An ostrogen-dependent model of breast cancer created by transformation of normal mammary epithelial cells. Breast Cancer Res. 9, R38 (2007). 17573968 10.1186/bcr1734 PMC1929103

[b39] Shehata M. *et al.* Phenotypic and functional characterisation of the luminal cell hierarchy of the mammary gland. Breast Cancer Res. 14, R134 (2012). 23088371 10.1186/bcr3334 PMC4053112

[b40] Visvader J. E. & Stingl J. Mammary stem cells and the differentiation hierarchy: current status and perspective. Genes Dev. 28, 1143–1158 (2014). 24888586 10.1101/gad.242511.114 PMC4052761

[b41] Honeth G. *et al.* Aldehyde dehydrogenase and estrogen receptor define a hierarchy of cellular differentiation in the normal human mammary epithelium. Breast Cancer Res. 16, R52 (2014). 24887554 10.1186/bcr3663 PMC4095680

[b42] Arendt L. M. *et al.* Human breast progenitor cell numbers are regulated by WNT and TBX3. PLoS ONE 9, 1–14 (2014). 10.1371/journal.pone.0111442 PMC4211891 25350852

[b43] Gupta P. B. *et al.* Stochastic state transitions give rise to phenotypic equlibrium in populations of cancer cells. Cell 146, 633–644 (2011). 21854987 10.1016/j.cell.2011.07.026

[b44] Shoker B. S. *et al.* Estrogen receptor-positive proliferating cells in the normal and precancerous breast. Am. J. Pathol. 155, 1811–1815 (1999). 10595909 10.1016/S0002-9440(10)65498-3 PMC1866935

[b45] Moses H. & Barcellos-Hoff M. H. TGF-β biology in mammary development and breast cancer. Cold Spring Harb. Perspect. Biol. 3, a003277 (2011). 20810549 10.1101/cshperspect.a003277 PMC3003461

[b46] Hansen A. G. *et al.* ALCAM/CD166 is a TGF-β-responsive marker and functional regulator of prostate cancer metastasis to bone. Cancer Res. 74, 1404–1415 (2014). 24385212 10.1158/0008-5472.CAN-13-1296 PMC4149913

[b47] Cuzick J. *et al.* Selective oestrogen receptor modulators in prevention of breast cancer: an updated meta-analysis of individual participant data. Lancet 381, 1827–1834 (2013). 23639488 10.1016/S0140-6736(13)60140-3 PMC3671272

[b48] de Assis S. *et al.* High-fat or ethinyl-oestradiol intake during pregnancy increases mammary cancer risk in several generations of offspring. Nat. Commun. 3, 1053–1061 (2012). 22968699 10.1038/ncomms2058 PMC3570979

[b49] Cowper-Sal-lari R. *et al.* Breast cancer risk-associated SNPs modulate the affinity of chromatin for FOXA1 and alter gene expression. Nat. Genet. 44, 1191–1200 (2012). 23001124 10.1038/ng.2416 PMC3483423

[b50] Laping N. J. *et al.* Inhibition of transforming growth factor (TGF)-β1-induced extracellular matrix with a novel inhibitor of TGF-β1 type I receptor kinase activity: SB-431542. Mol. Pharmacol. 62, 58–64 (2002). 12065755 10.1124/mol.62.1.58

[b51] Gellibert F. *et al.* Identification of 1,5-naphthyridine derivatives as a novel series of potent and selective TGF-β type I receptor inhibitors. J. Med. Chem. 47, 4494–4506 (2004). 15317461 10.1021/jm0400247

[b52] Ichida J. K. *et al.* A small-molecule inhibitor of TGF-β signaling replaces *Sox2* in reprogramming by inducing *Nanog*. Cell Stem Cell 5, 491–503 (2009). 19818703 10.1016/j.stem.2009.09.012 PMC3335195

[b53] Ince T. A. *et al.* Transformation of different human breast epithelial cell types leads to distinct tumor phenotypes. Cancer Cell 12, 160–170 (2007). 17692807 10.1016/j.ccr.2007.06.013

[b54] Soule H. D. *et al.* Isolation and characterization of a spontaneously immortalized human breast epithelial cell line, MCF-10. Cancer Res. 50, 6075–6086 (1990). 1975513

[b55] Briand P., Petersen O. W. & van Deurs B. A new diploid nontumorigenic human breast epithelial cell line isolated and propagated in chemically defined medium. In Vitro Cell. Dev. Biol. 23, 181–188 (1987). 3558253 10.1007/BF02623578

[b56] Pasic L. *et al.* Sustained activation of the HER1-ERK1/2-RSK signaling pathway controls myoepithelial cell fate in human mammary tissue. Genes Dev. 25, 1641–1653 (2011). 21828273 10.1101/gad.2025611 PMC3182019

[b57] Kiyono T. *et al.* Both Rb/p16INK4a inactivation and telomerase activity are required to immortalize human epithelial cells. Nature 396, 84–88 (1998). 9817205 10.1038/23962

[b58] Counter C. M. *et al.* Dissociation among *in vitro* telomerase activity, telomere maintenance, and cellular immortalization. Proc. Natl Acad. Sci. USA 95, 14723–14728 (1998). 9843956 10.1073/pnas.95.25.14723 PMC24516

[b59] Hines W. C., Yaswen P. & Bissell M. J. Modelling breast cancer requires identification and correction of a critical cell lineage-dependent transduction bias. Nat. Commun. 6, 6927–6937 (2015). 25896888 10.1038/ncomms7927 PMC4411288

[b60] Dvinge H. & Bertone P. HTqPCR: high-throughput analysis and visualization of quantitative real-time PCR data in R. Bioinformatics 25, 3325–3326 (2009). 19808880 10.1093/bioinformatics/btp578 PMC2788924

[b61] Livak K. J. & Schmittgen T. D. Analysis of relative gene expression data using real-time quantitative PCR and the 2-2^-ΔΔ^Ct method. Methods 25, 402–408 (2001). 11846609 10.1006/meth.2001.1262

[b62] Rønnov-Jessen L., Celis J. E., van Deurs B. & Petersen O. W. A fibroblast-associated antigen: characterization in fibroblasts and immunoreactivity in smooth muscle differentiated stromal cells. J. Histochem. Cytochem. 40, 475–486 (1992). 1552184 10.1177/40.4.1552184

