# Supplementary material for: Propagation of oestrogen receptor-positive and oestrogen-responsive normal human breast cells in culture
Source: Nat Commun. 2015 Nov 13;6:8786. doi: 10.1038/ncomms9786 (PMC4660059; doi:10.1038/ncomms9786)
Supplement: Supplementary Information — Supplementary Figures 1-13 and Supplementary Table 1 [file ncomms9786-s1.pdf]

Supplementary Figure 1. Ks20.8 staining is independent of antibody supplier and ER staining when detected by immunofluorescence reflects ER/PR staining

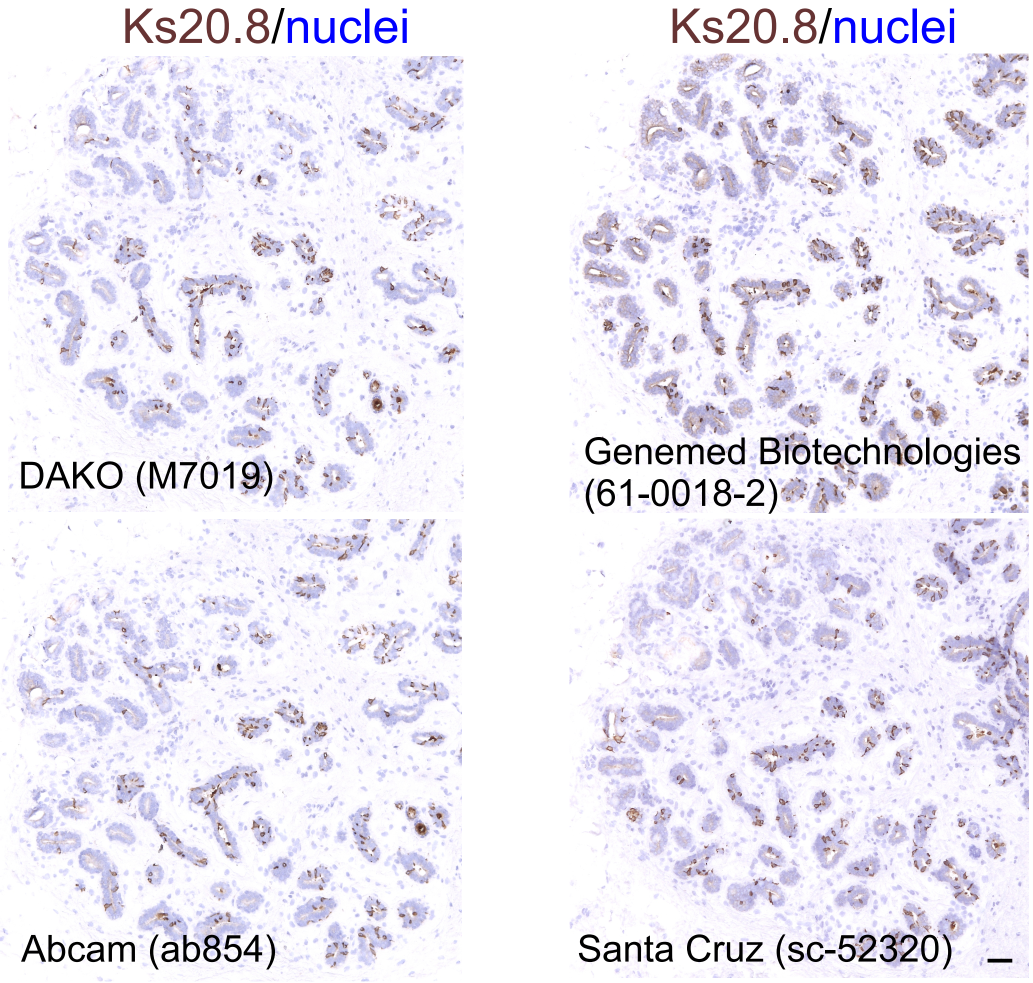

a

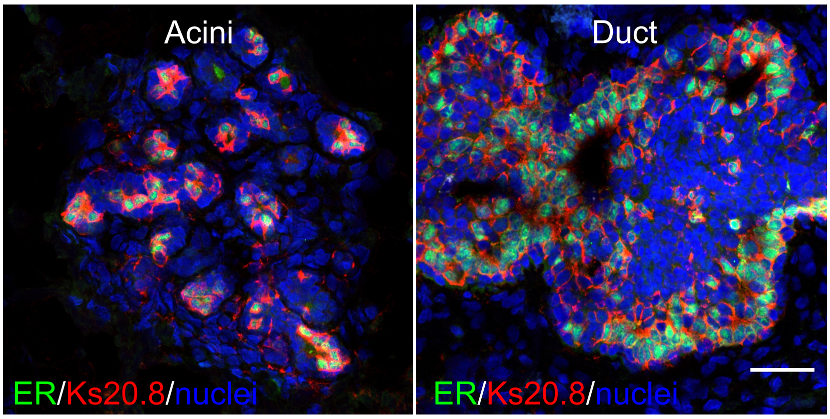

b

(a) Micrographs of serially sectioned human breast tissue immunoperoxidase stained with Ks20.8 purchased from four different suppliers (DAKO, Abcam, Genemed and Santa Cruz). All antibodies showed the same staining pattern. Nuclei are counterstained with hematoxylin. Bar = 50μm.(b) Multicolor imaging of cryostat sections from the same normal human breast tissue as used in figure 1b and stained with estrogen receptor (SP1; green), Ks20.8 (red), and nuclei (blue). ER protein localizes to essentially the same cells as Ks 20.8 and reflects ER/PR staining in tissues with high expression. Bar=25μm.

Supplementary Figure 2. Ks20.8 positive cells exhibit an elaborate differentiation program

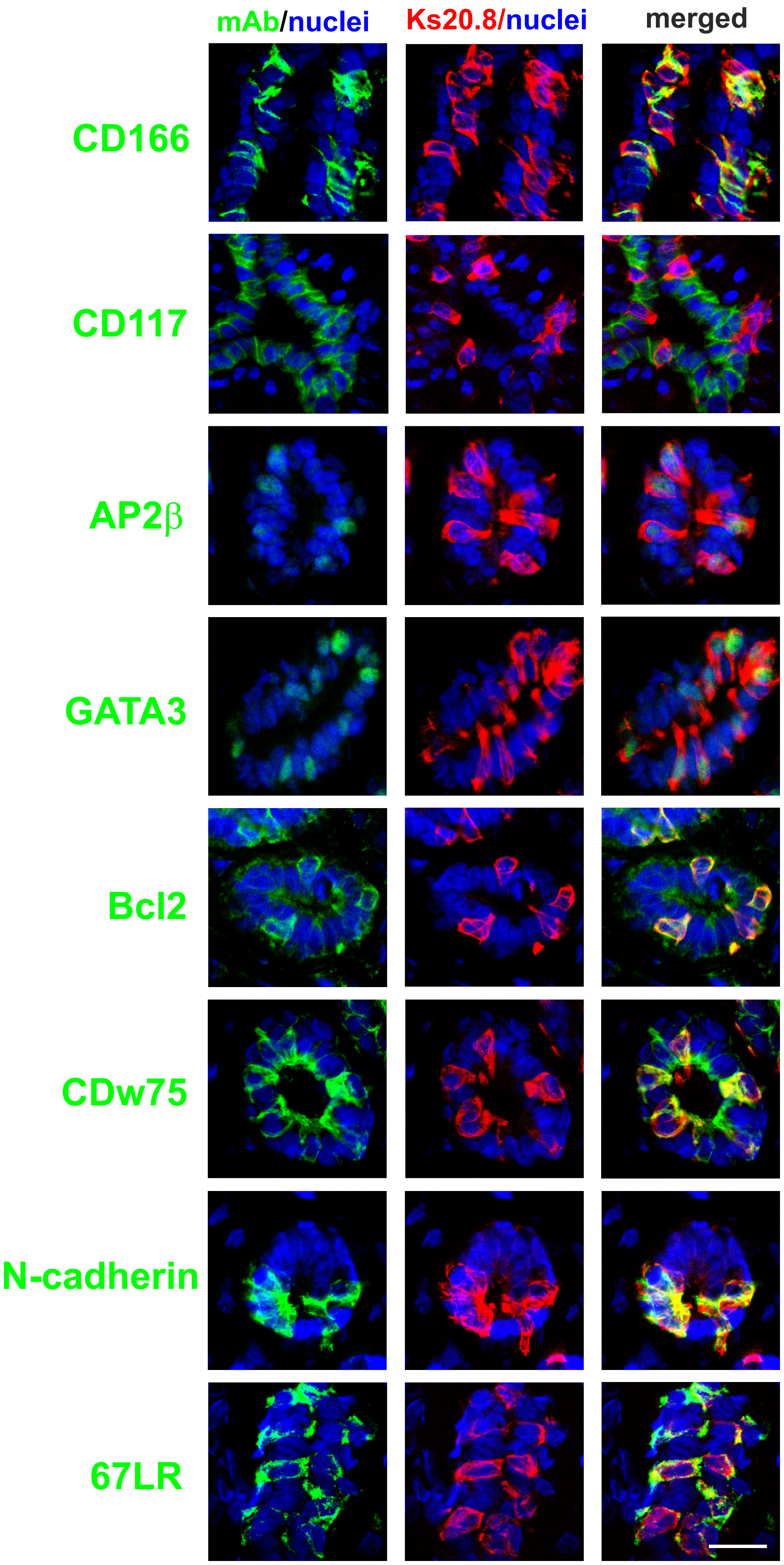

Multicolor imaging of cryostat sections from normal human breast tissue stained with selected mAbs (green) from a library, Ks20.8 (red), and nuclei (blue). Note that selected markers except CD117 are colocalized with Ks20.8. Bar=25μm.

Supplementary Figure 3. Representative FACS gating strategy of sorted cells

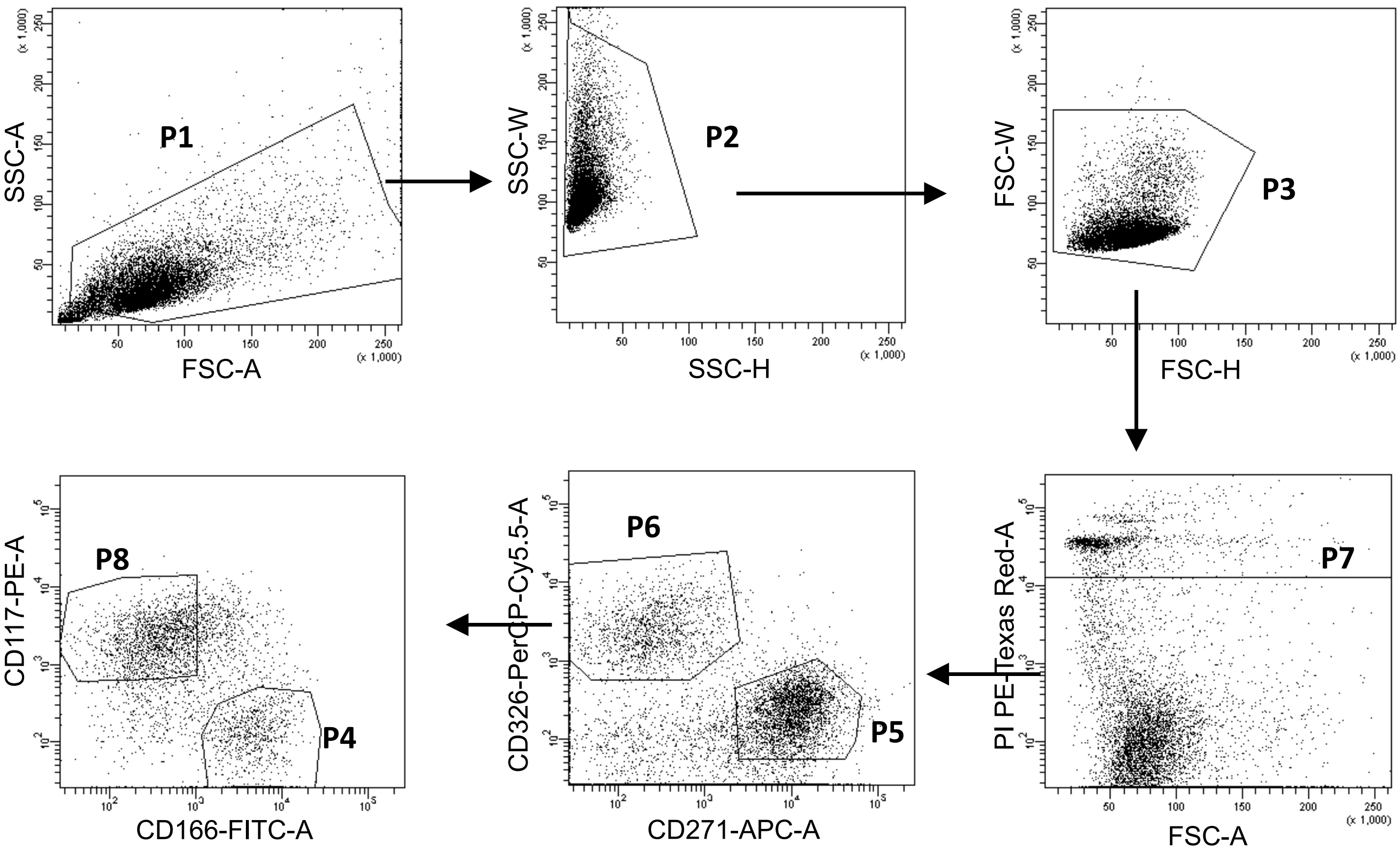

Tube: P790\

Population	#Events	%Parent	%Total
All Events	50,000		100.0
P1	44,803	89.6	89.6
P2	44,353	99.0	88.7
P3	44,284	99.8	88.6
P7	38,063	86.0	76.1
P5	15,144	39.8	30.3
P6	7,106	18.7	14.2
P4	1,737	24.4	3.5
P8	3,319	46.7	6.6

Cells were gated on forward scatter (FSC) and side scatter (SSC) to remove debris and select for single cells. Propidium iodide was used to select for live cells which were then further gated for luminal CD326^{high} cells (P6) and basal CD271^{high} cells (P5). CD326^{high} luminal cells were further sub-gated as non-overlapping gates of CD166^{high} (P4) and CD117^{high}(P8).

Supplementary Figure 4. Hormone receptor expression is restricted essentially to the Ks20.8^{pos} compartment

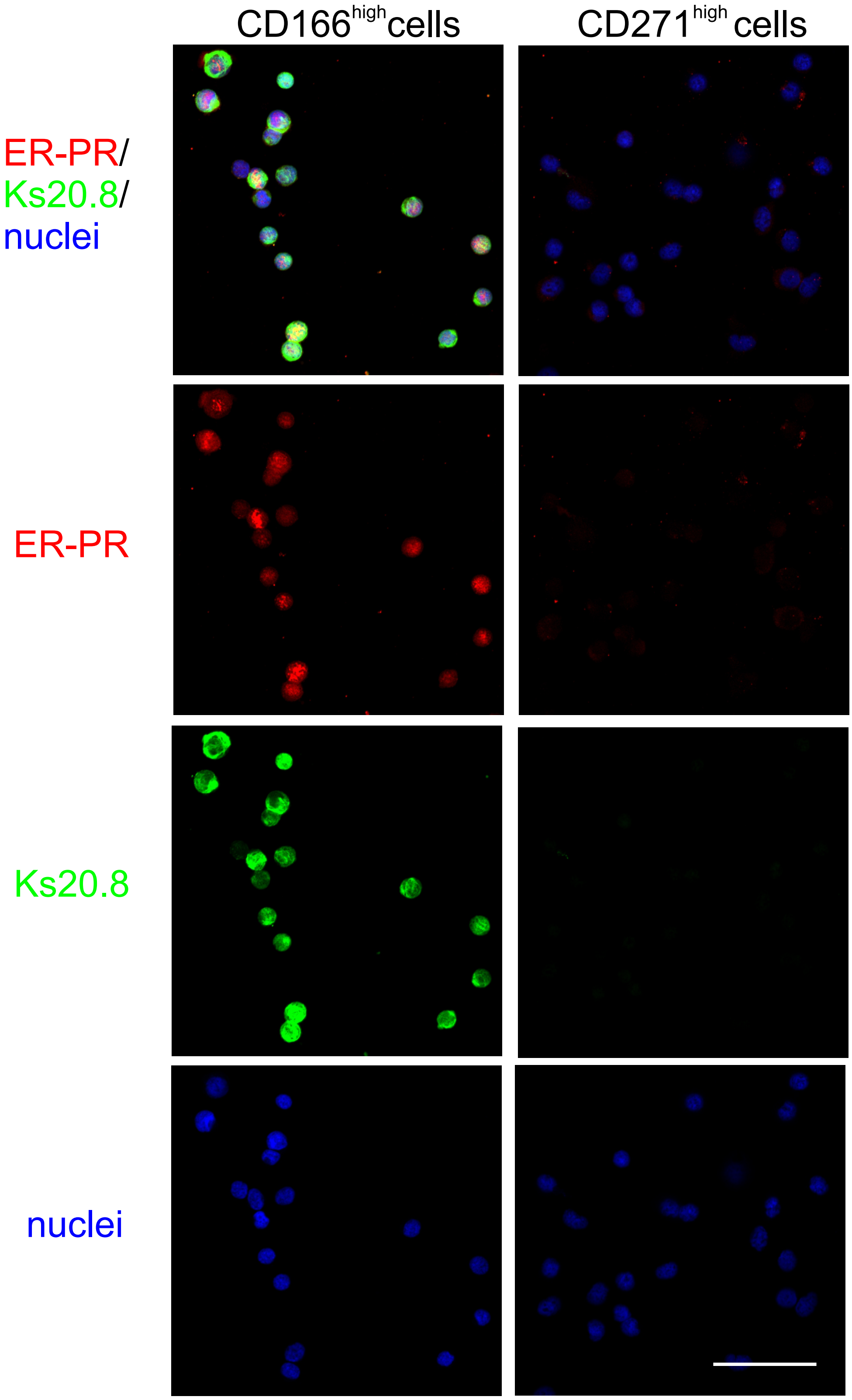

Multicolor imaging of smears of normal human CD166^{high}/CD117^{low} (CD166^{high} cells) and basal cells (CD271^{high} cells) purified by FACS and stained with ER-PR (SP1, SP2; red), Ks20.8 (green) and nuclei (blue). The vast majority of ER-PR^{pos} cells are contained within the Ks20.8^{pos} compartment. Bar=50 μm.

Supplementary Figure 5. Profiling of basal cells, CD117^{high} and CD166^{high} cells

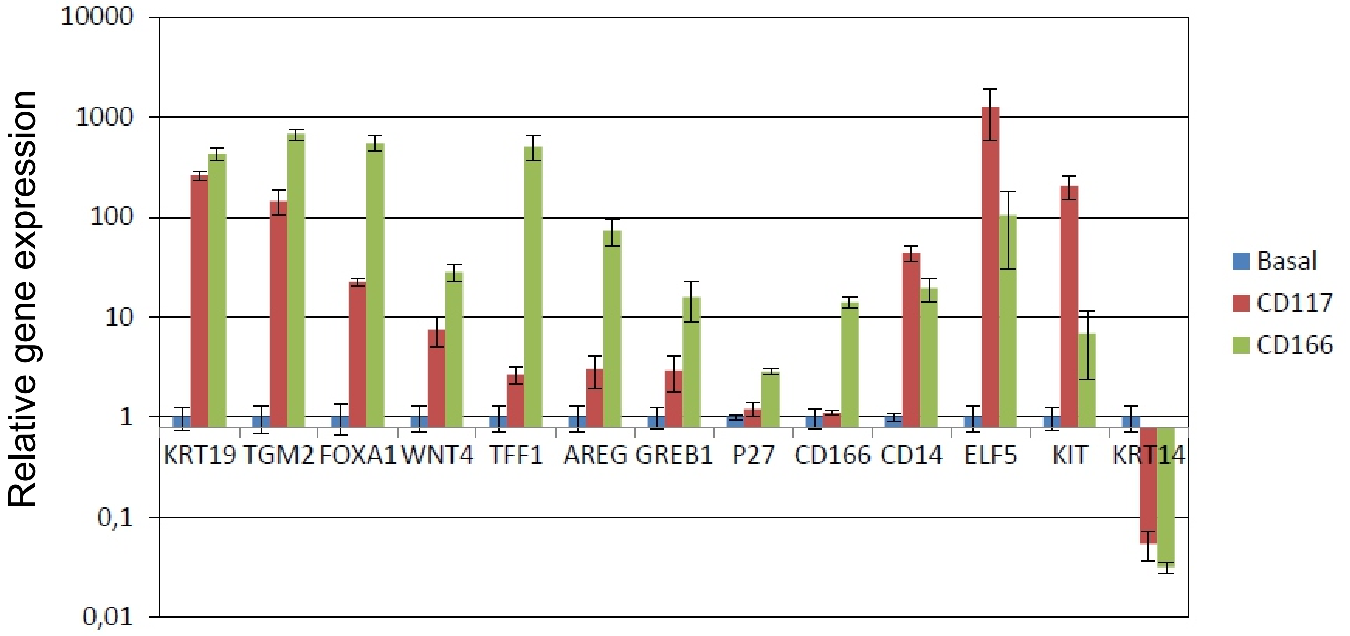

Bar graph representing qRT-PCR analysis of additional gene expression profiles in sorted basal, CD117^{high}, and CD166^{high} cells from three different biopsies.
Each gene expression level was normalized by genomic mean of four reference gene expressions and then compared to normalized gene expression levels in basal cells.
Error bars represent SEM of three biological samples which were each run in triplicate by qPCR.

Supplementary Figure 6. Five out of six biopsies show a distinct CD166^{high} population

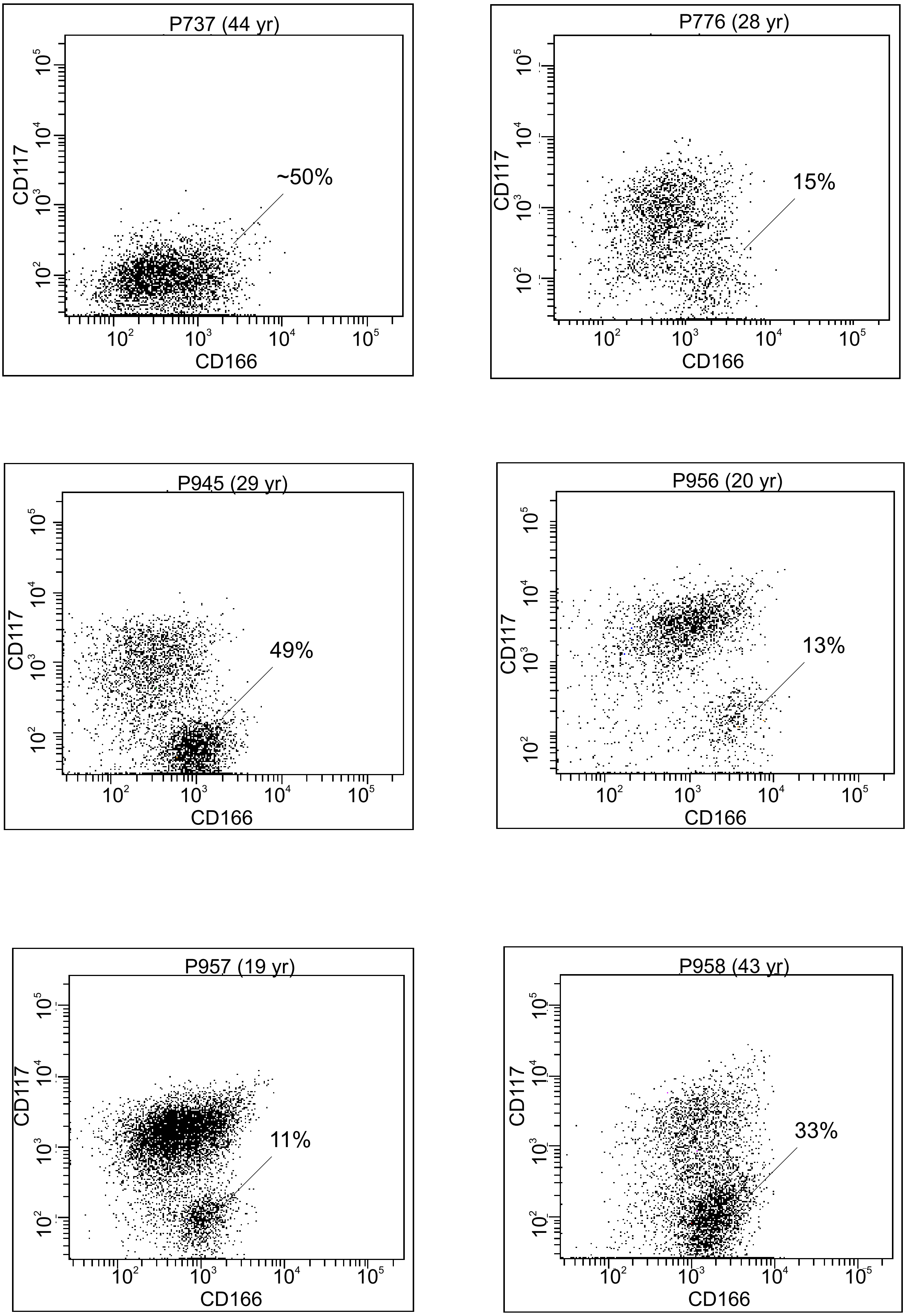

Representative FACS diagrams of uncultured HBEC from six biopsies sorted with CD166 and CD117.

Supplementary Figure 7. Representative FACS profiles illustrating the separation of 67LR^{high}/CD166^{high} cells from uncultured HBECs

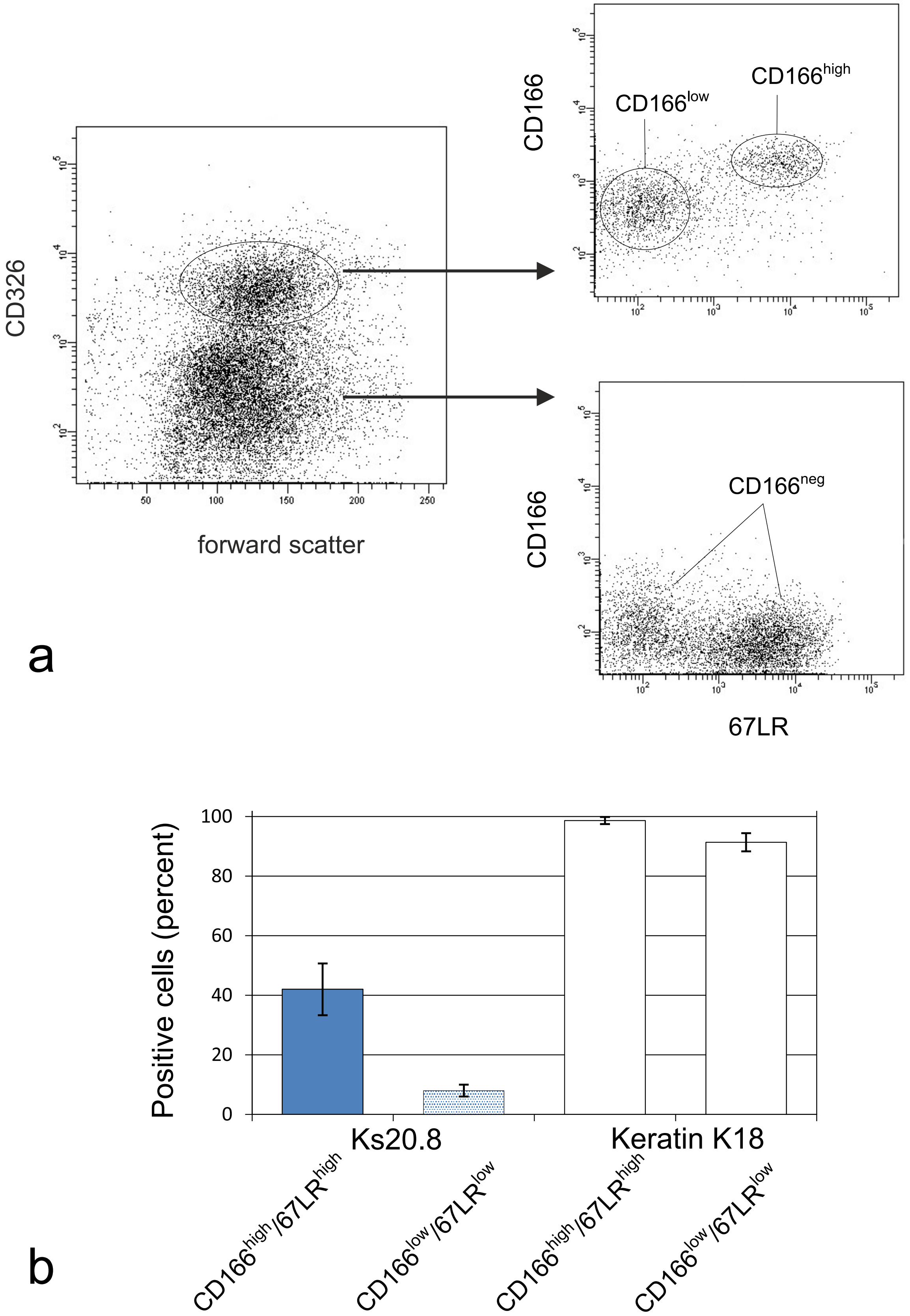

(**a**) A single cell suspension of primary breast epithelial cells stained with CD326, CD166 and 67LR followed by flow cytometry. Ks20.8^{pos} HBEC sorted with the CD166^{high}/67LR^{high} gate. (**b**) Smears of FACS sorted cells stained with Ks20.8 (left) or keratin K18 (right). Frequency of positive cells (n = 3x50 cells per slide ±SD).

Supplementary Figure 8. PCR array of human estrogen receptor signaling

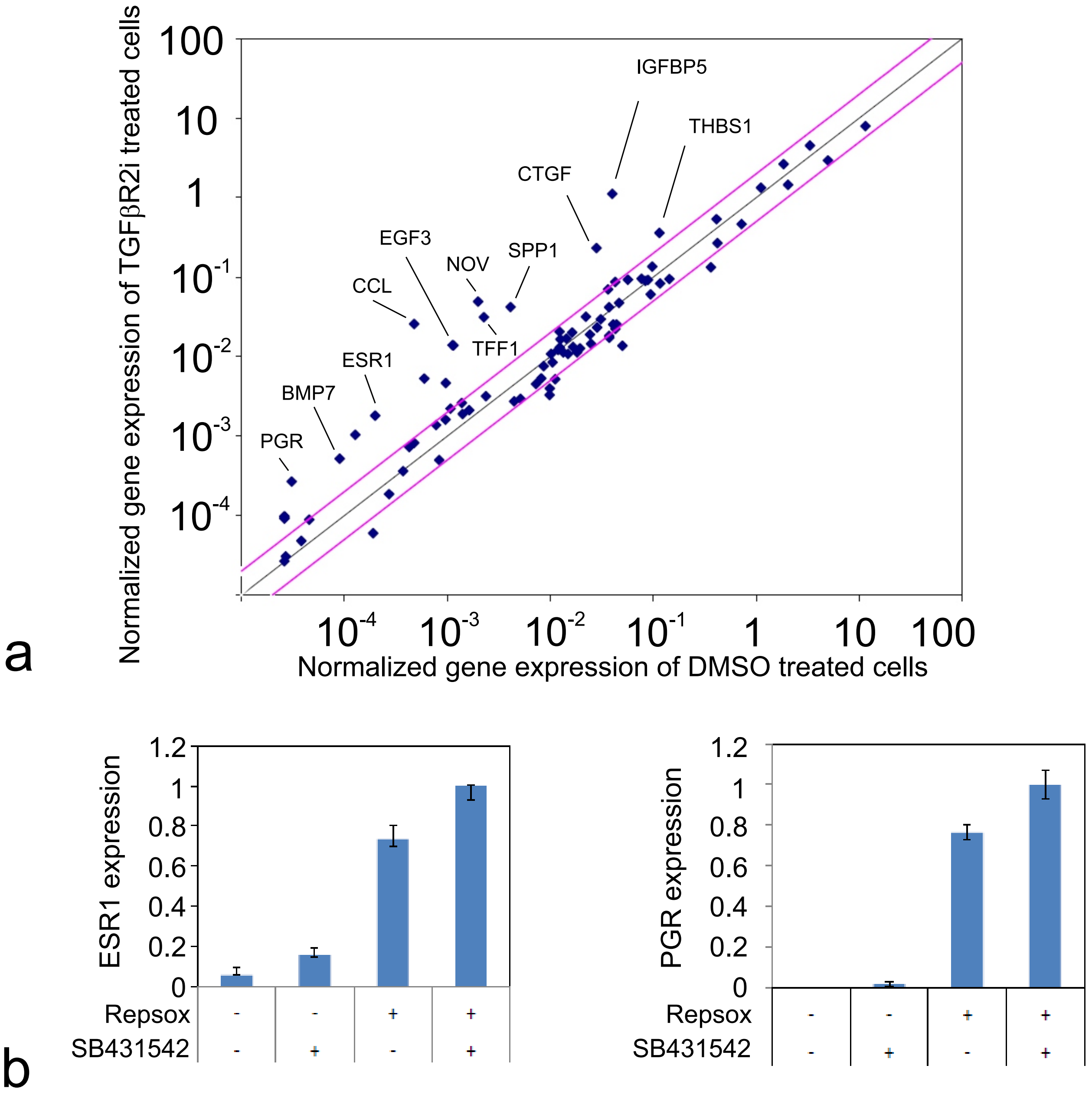

(**a**) Using a commercially available PCR array containing 84 genes involved in ER activation and its response, qRT-PCR was conducted with RNA samples of ER^{pos}HBEC with or without TGFβR2i. Among them, 20 genes (some of which are labeled in the plot) were highly expressed in the presence of TGFβR2i. Scatter plot graph shows the expression level of each gene in TGFβR2i treated $CD166^{high}$ cells versus DMSO treated $CD166^{high}$ cells. The black line indicates no difference between samples, while the pink lines indicate a two-fold change in gene expression. Two biological replicates were run in qPCR with duplicates of each. (**b**) qRT-PCR of ESR1 and PGR expression in hTERT/shp16 transduced ER^{pos} HBECs exposed to vehicle(DMSO), SB431542 alone, Repsox alone or TGFβR2i for 7 days. Note that the highest levels of expression were obtained in presence of TGFβR2i. Colored bars indicate the fold difference of the relative gene expression and error bars represent SD of triplicates in qPCR.

Supplementary Figure 9. Full scale Western blots underlying Figure 4d

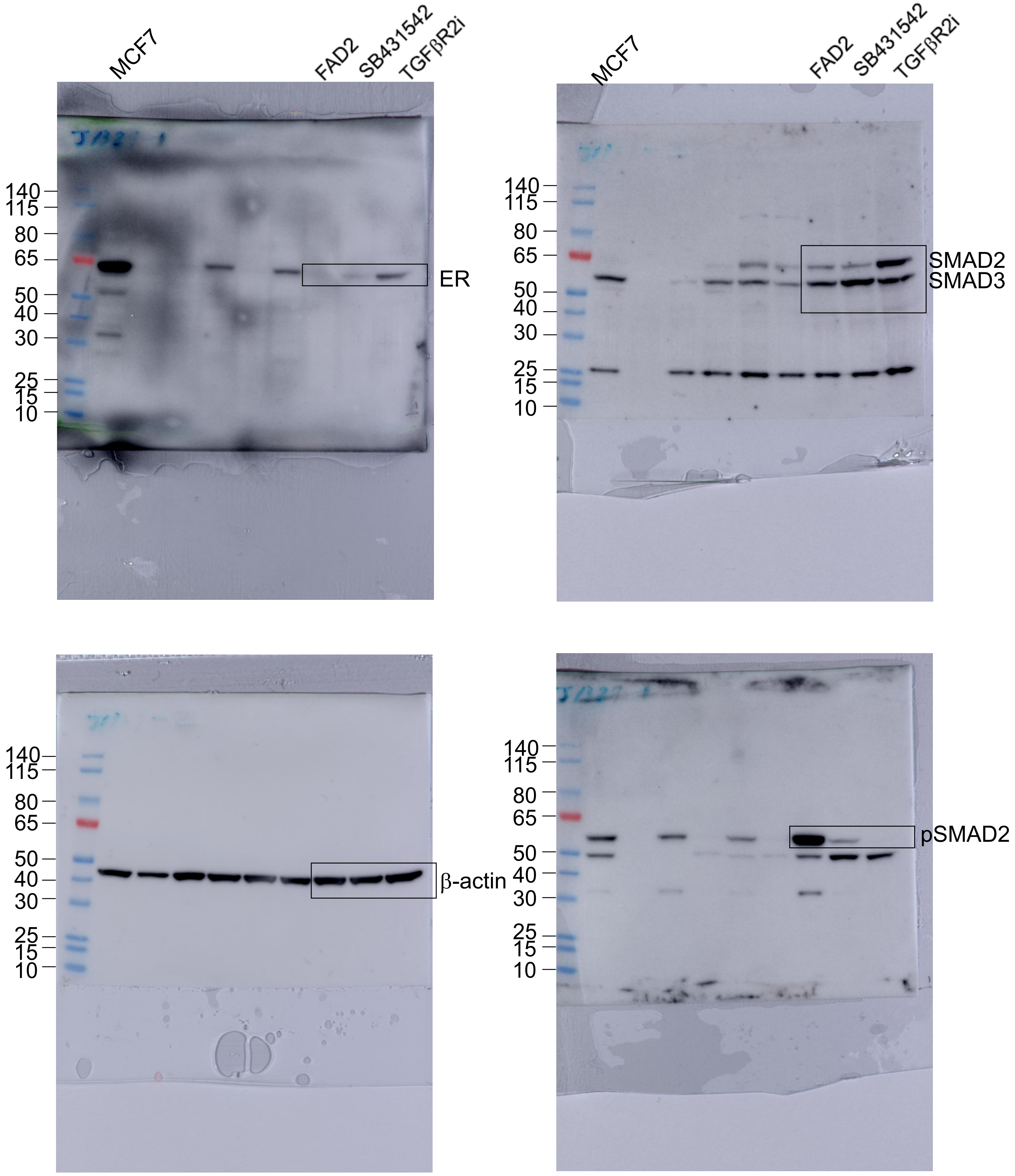

The frames indicate areas of the original Western blots selected for making the composite Figure 4d.

Supplementary Figure 10. TGFβRII expression in ER^{pos} HBEC

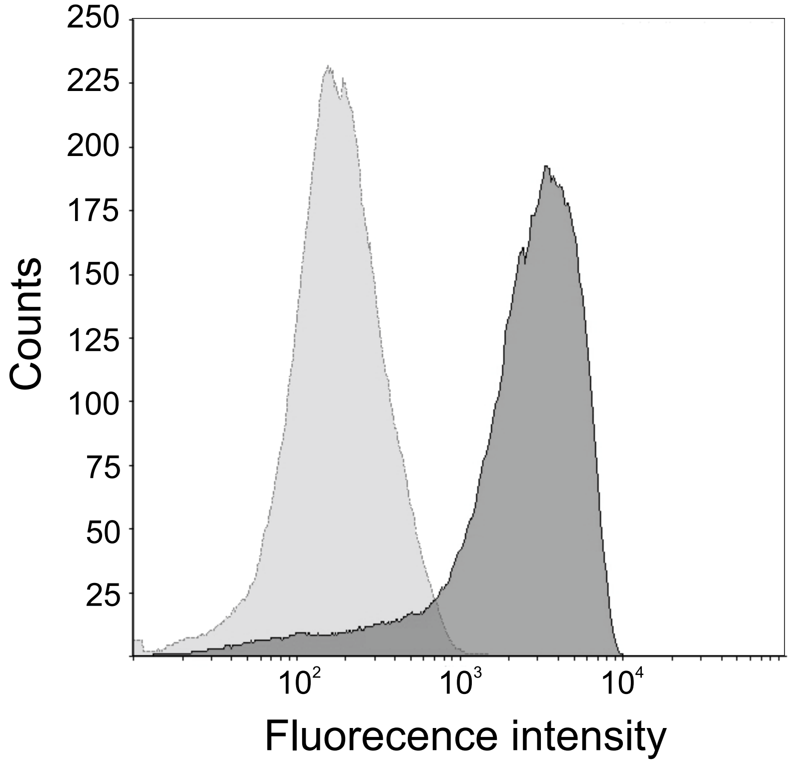

FACS analysis of TGFβRII surface expression in short-term cultured ER^{pos} HBEC. FITC-TGFβRII (dark grey). FITC conjugated antibody in the absence of TGFβRII primary antibody (light grey).

Supplementary Figure 11. hTERT/shp16 transduced cells remain ER^{pos} and luminal-like

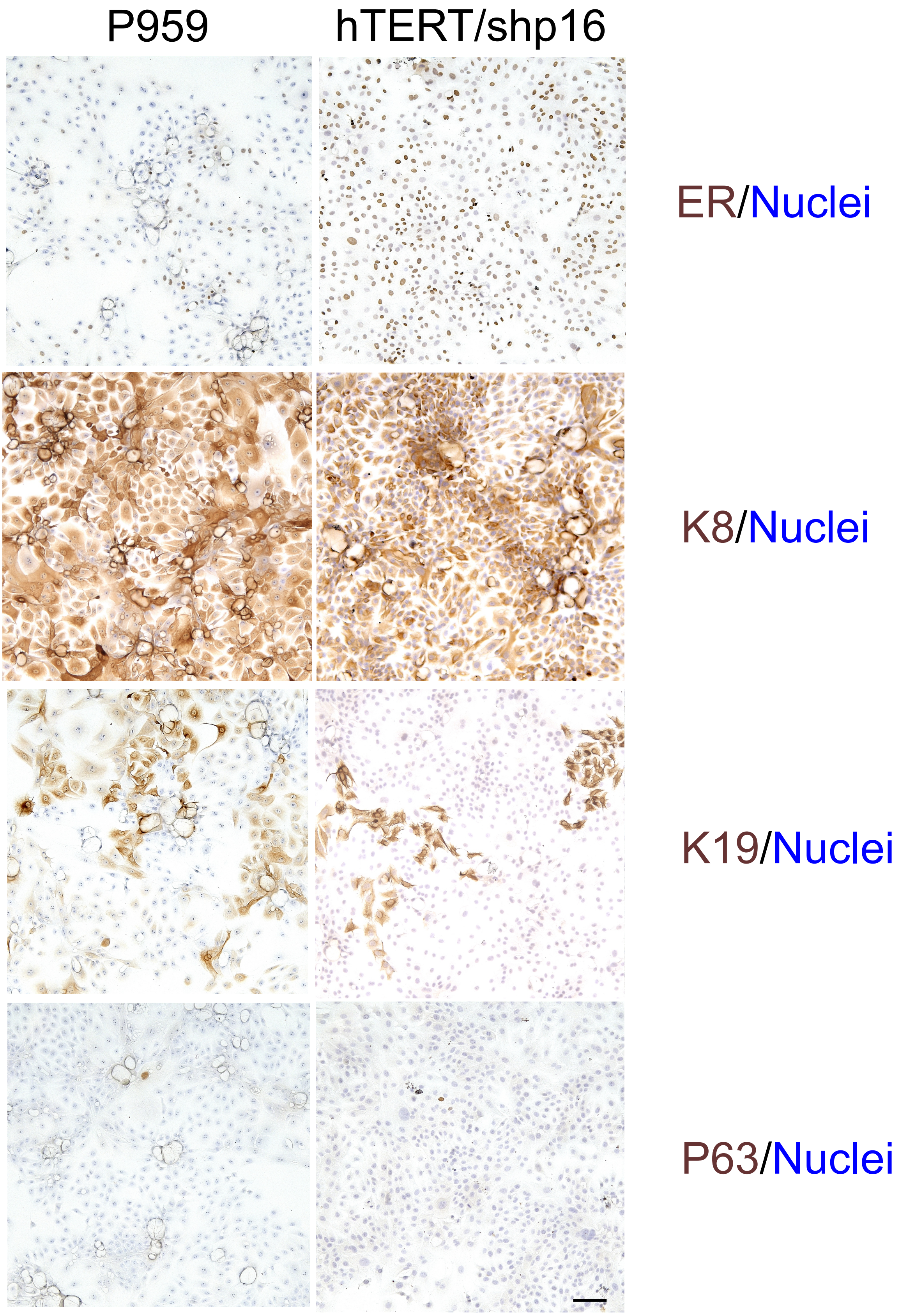

Immunoperoxidase staining of finite life span (left column) and hTERT/shp16 transduced (right column) ER^{pos}HBEC with ER (SP1 prediluted), keratin K8 and K19 and P63. Cells were counterstained with hematoxylin. (Bar = 100μm)

Supplementary Figure 12. Estrogen induced growth is completely abolished by pure antiestrogen

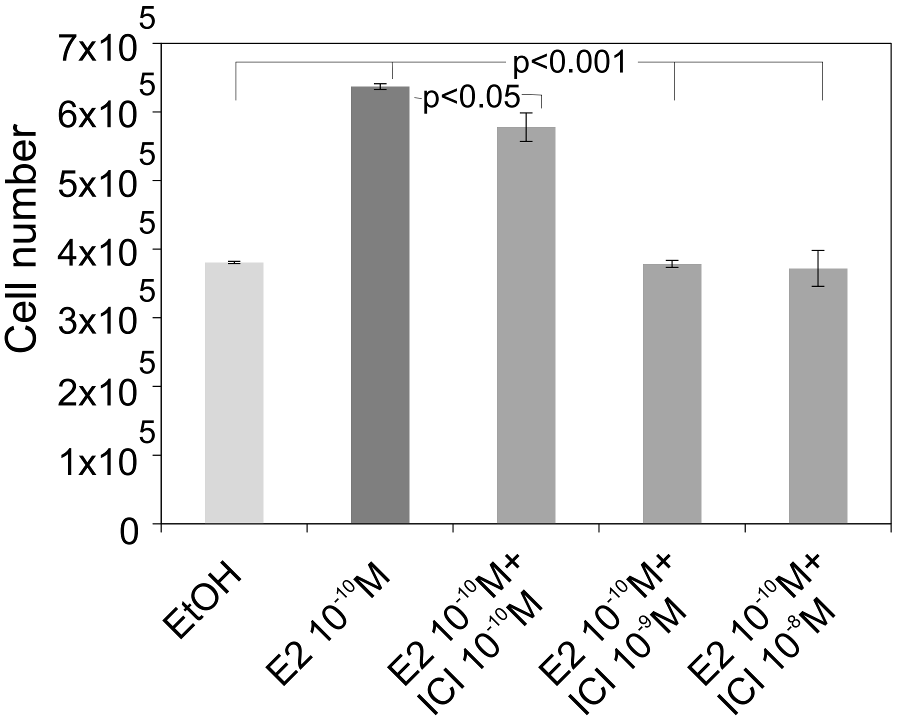

ER^{pos} HBEC with extended lifespan (hTERT/shp16) exposed for six days to vehicle (EtOH), E2 or E2 plus increasing concentrations of ICI182,780/Fulvestrant/Faslodex. Note that the pure antiestrogen abolishes the growth promoting effect of estrogen. Significance was assessed by ANOVA followed by Tukey´s HSD test.
Error bars represent SD of duplicates in EtOH and E2 and triplicates in ICI treated samples.

Supplementary Figure 13. Augmented effect of estrogen on ER^{pos}HBEC by long-term exposure and omission of EGF

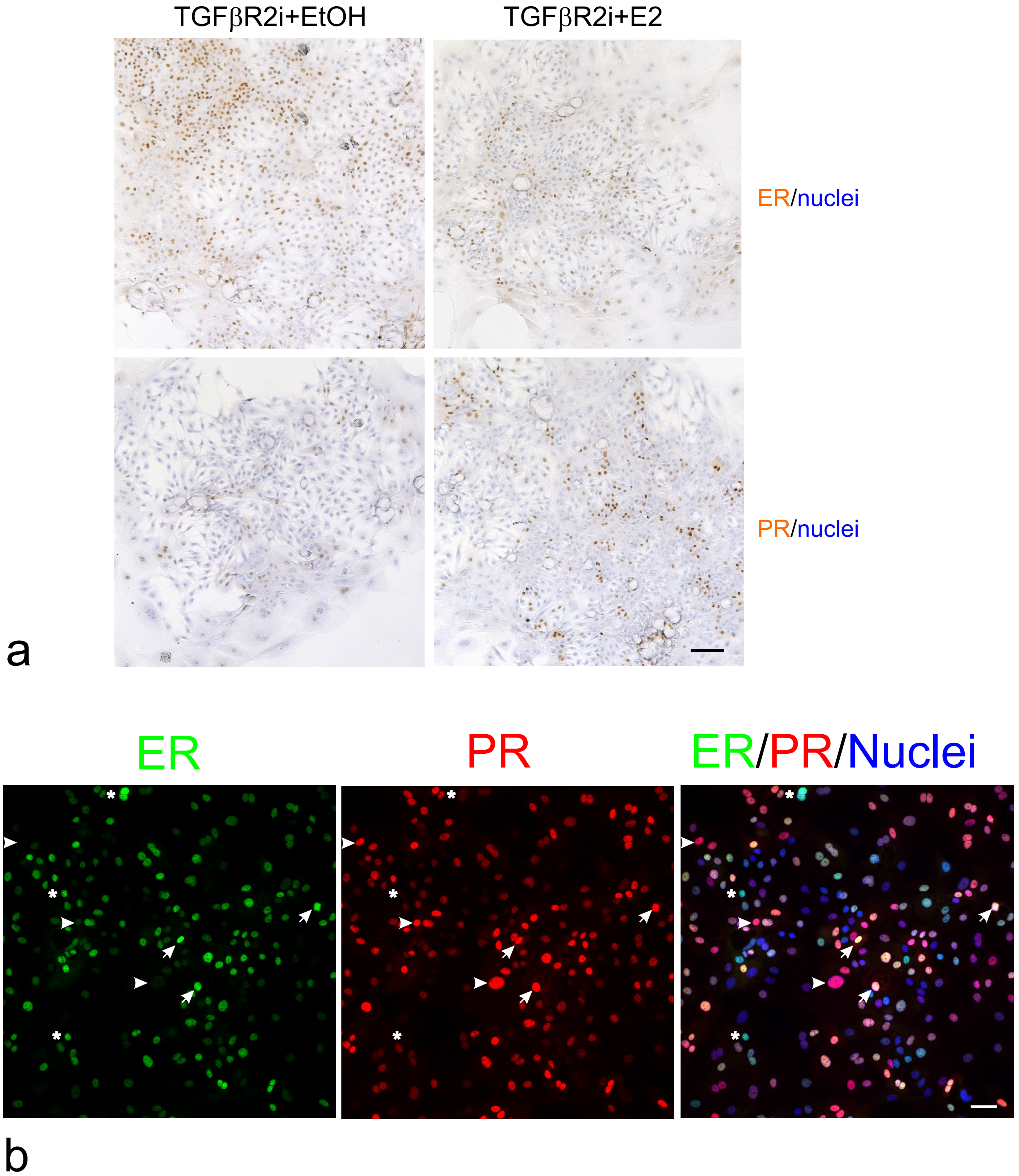

(**a**) Immunoperoxidase stainings of ER^{pos}HBEC kept on TGFβR2i and in the presence of estrogen for two passages prior to omission of estrogen in the third passage (left column). Note the marked difference in staining with ER (SP1, prediluted) and PR (SAN27). Cells were counterstained with hematoxylin. (Bar = 100μm). (**b**) Multicolor imaging of ER^{pos} HBEC in the presence of estrogen stained with ER (SP1; green), PR (PgR636; red) and nuclei (blue). Cells are either ER^{pos} (asterix), PR^{pos} (arrowhead), ER^{pos}/PR^{pos} (arrows), or ER^{neg}/PR^{neg} (not labeled). (Bar = 50μm).

Supplementary Table 1. List of primers used for qRT-PCR

Gene symbol	Assay ID	Category
ACTA2	Hs00426835_g1	Basal marker
CD200	Hs01033303_m1	Basal marker
CD271	Hs00609977_m1	Basal marker
IGFBP3	Hs00365742_g1	Basal marker
KRT14	Hs00265033_m1	Basal marker
KRT5	Hs00361185_m1	Basal marker
MME	Hs00153510_m1	Basal marker
NGR1	Hs00247620_m1	Basal marker
SNAI2/SLUG	Hs00950344_m1	Basal marker
ST3GAL2	Hs00199480_m1	Basal marker
AREG	Hs00950669_m1	Luminal marker
BIM	HS00708019_s1	Luminal marker
CD166	Hs00977640_m1	Luminal marker
CDKN1B/P27	Hs00153277_m1	Luminal marker
ESR1	Hs00174860_m1	Luminal marker
FOXA1	Hs00270129ml	Luminal marker
GATA3	Hs00231122_m1	Luminal marker
GREB1	Hs00536409_m1	Luminal marker
ID2	Hs04187239_m1	Luminal marker
KRT18	Hs02827483_g1	Luminal marker
KRT19	Hs00761767_s1	Luminal marker
KRT8	Hs01595539_g1	Luminal marker
MYB	Hs00920556_m1	Luminal marker
NGRN	Hs04185079_g1	Luminal marker
PGR	Hs01556702_m1	Luminal marker
SORT1	Hs00361760_m1	Luminal marker
TFF1	Hs00907239_m1	Luminal marker
TGM2	Hs00190278_m1	Luminal marker
WNT4	Hs01573504_m1	Luminal marker
DPP4	HS00175210_m1	Luminal progenitor marker
ALDH1A3	Hs00167476_m1	Luminal progenitor marker
CD14	Hs00169122_g1	Luminal progenitor marker
CYP24A1	Hs00167999_m1	Luminal progenitor marker
DAPP1	Hs00183937_m1	Luminal progenitor marker
ELF5	Hs01063022_m1	Luminal progenitor marker
KIT	Hs00174029_m1	Luminal progenitor marker
KRT15	Hs00267035_m1	Luminal progenitor marker
NCALD	Hs00230737_m1	Luminal progenitor marker
PIGR	Hs00922561_m1	Luminal progenitor marker
Sox9	Hs01001343_g1	Luminal progenitor marker
GAPDH	Hs02758991_g1	Reference
HPRT1	Hs99999909_m1	Reference
TBP	Hs00427621_m1	Reference
TFRC	Hs00951083_m1	Reference
